# Single-nucleus transcriptomics resolves multiple fate dynamics between inflorescence meristem and primary stem

**DOI:** 10.1126/sciadv.aee2988

**Published:** 2026-06-19

**Authors:** Sebastián Moreno-Ramírez, Martin O. Lenz, Elliot M. Meyerowitz, James C. W. Locke, Henrik Jönsson

**Affiliations:** ^1^ Sainsbury Laboratory, University of Cambridge, Bateman Street, Cambridge CB2 1LR, UK.; ^2^ Cambridge Advanced Imaging Centre, Downing Site, University of Cambridge, Cambridge CB2 3DY, UK.; ^3^ Division of Biology and Biological Engineering and Howard Hughes Medical Institute, 156-29, California Institute of Technology, Pasadena, CA 91125, USA.; ^4^ Centre for Environmental and Climate Science, Lund University, S-221 00 Lund, Sweden.

## Abstract

The inflorescence meristem (IM) of flowering plants is a stem cell niche that is the source of aboveground organs such as stems and flowers. Although transcriptional profiling has elucidated some cell types within this tissue, the transcriptional dynamics of differentiation from stem cells into the diverse cell types in these organs remain unknown. We used single-nucleus RNA sequencing to characterize the transcriptional landscape of the IM, linking shoot stem cells to early differentiation cell types. Although our analysis of previously known domains uncovered the role of *GH3* gene family members in meristematic activity and phyllotaxis, we also identified unknown transcriptional patterns, including early cortex patterning within developing primordia. Trajectory inference analysis revealed the dynamics of the S-G_2_-M cell cycle phases, as well as gene expression programs driving differentiation toward specialized cell types such as early primordia, cortex, cambium, xylem, and phloem. Collectively, our findings advance the understanding of the cell fate transcriptional dynamics shaping shoot organ development.

## Teaser

snRNA-seq reveals shoot stem cell heterogeneity and traces their differentiation into primary stem cell types.

## INTRODUCTION

In contrast to many animals, whose organogenesis is confined to embryonic or larval stages, plants generate organs throughout their life span. This continuous organogenesis is orchestrated by stem cells situated within specialized regions known as meristems (*1*). The shoot apical meristem (SAM), located at each shoot apex, is accountable for the formation of aboveground organs. Meristem identity shifts throughout plant development. As the plant transitions to a reproductive stage, the vegetative SAM converts into an inflorescence meristem (IM), which orchestrates the development of flowers and stems (*2*).

In angiosperms, IM stem cells reside in two overlapping zones: the central zone (CZ) and the organizing center (OC), the first apical and the second only in inner layers (*3*). These stem cell reservoirs displace daughter cells outwardly toward the periphery and inner stem, where they will differentiate into the various cell types observed in aerial organs (*4*). Most CZ lineages within the IM are characterized by anticlinal division, ensuring isolated cell lineages within the epidermal layer and subepidermal layers (*5*). Conversely, OC lineages undergo both periclinal and anticlinal divisions (*5*), forming the diverse compendium of cell types present in inner layers of the primary stem such as cortex, rib zone, and vascular bundles (*6*). The mechanisms and transcriptional dynamics linking shoot stem cells to the diverse cell types observed in the primary stem remain largely unknown.

In *Arabidopsis thaliana*, the CZ is positioned centrally in the IM and is demarcated by the expression of the *CLAVATA 3* (*CLV3*) peptide-encoding gene. *CLV3* is expressed in the epidermis and in inner layers where it overlaps with cells expressing the RNA for the transcription factor (TF) WUSCHEL (WUS), which defines the OC domain (*7*, *8*). Beyond these stem cell pools, other marker genes and/or characteristic morphological domains demarcate various cell types in the IM, such as the boundary domain (BD) in the boundary between new floral primordia and the meristem (*9*), an auxin-responsive domain marking floral primordia initiation (*10*) and peripheral zone (PZ) surrounding the OC and CZ. Genes whose expression is characteristic of vascular cell types in the stem are also expressed in specific domains within the IM, defining procambial strands during primordia formation (*6*) or exhibiting an internalized linear pattern starting from the rib zone that exists basal to the OC (*11*). Although some cell types within the stem and flowers such as procambium have been identified to originate within the meristem, whether there are other cell types defined within the meristematic domains is unknown.

In recent years, single-cell RNA sequencing (scRNA-seq) approaches have been used to characterize cellular heterogeneity within the shoot apex across diverse plant species such as *Arabidopsis* (*12*, *13*), rice (*14*), *Populus* (*15*), maize (*16*, *17*), and tomato (*18*). These studies have expanded our understanding and resolution of the cellular landscapes previously characterized by fluorescence-activated cell sorting (FACS sorting) and translating ribosome affinity purification (TRAP) within the SAM (*19*–*21*). Although these approaches have offered critical insights into the diversity of cell types in the vegetative apex (*12*) and floral meristem (*22*), the gene expression patterns and gene regulatory networks (GRNs) that characterize the developmental transitions between IM and differentiating stem cell types remain to be revealed.

To deepen our understanding of shoot stem cell populations, several studies have used *Arabidopsis* mutant *ap1*-*1 cal-1* characterized by overproliferation of arrested secondary shoot meristems at the IM (*13*, *20*, *22*), which has allowed the enrichment of shoot stem cells. However, the distinct morphological profile, including developmental arrest, compared to wild type may limit the full reconstruction of differentiation trajectories from shoot stem cells to the diverse array of specialized cell types within the primary stem. Further studies focusing on wild-type contexts could help to bridge this gap and provide a comprehensive IM atlas with its multiple differentiation processes.

In this study, we used a single-nucleus RNA sequencing (snRNA-seq) approach from finely dissected wild-type IMs to unravel the transcriptional heterogeneity and cell differentiation processes within the IM. Our analysis unveiled transcriptional profiles for most previously known cell types such as BD, procambium, xylem parenchyma, early primordia (EP), epidermis, S phase and G_2_-M phase, and inner stem cells. By identifying differentially expressed genes (DEGs) in each cluster, we validated the expression of *GH3* auxin conjugation genes during EP formation and their role in maintaining shoot meristem homeostasis. In addition, we observed that cortex is defined along with vasculature during primordia formation. Furthermore, trajectory analysis allowed us to reconstruct cell cycle gene expression dynamics in the SAM and infer transcriptional landscape from shoot stem cells to diverse differentiated cell identities such as the cortex, xylem, phloem, cambium, and EP. This advances our understanding of the cellular and transcriptional correlates of cell fate acquisition from SAM stem cells.

## RESULTS

### Gene expression heterogeneity captured diverse biological functions in the IM

**Fig. 1.**
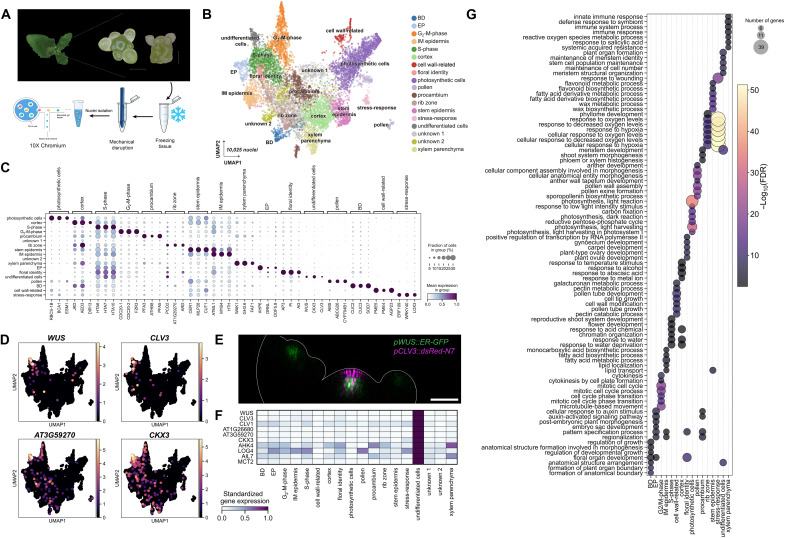
snRNA-seq resolves cellular heterogeneity within the *A. thaliana* IM. (**A**) Schema with the general workflow used for single-nucleus transcriptomic analysis from dissected *A. thaliana* IMs. Floral meristems with distinct sepals were removed (>stage 3). SAMs were frozen and then mechanically disrupted by pestle homogenization. Nuclei were FACS sorted before loading onto 10X Chromium chips. Scale bar, 100 μm. (**B**) UMAP and projection of cellular heterogeneity in IM tissue. (**C**) Dot plot showing selected marker genes for each cell cluster. The color bar indicates the gene-normalized gene expression. Dot size indicates the fraction of cells expressing marker genes. (**D**) UMAP highlighting cells expressing stem cell–related marker genes such as *WUS*, *CLV3*, *AT3G59270*, and *CKX3*. Normalized gene expression values are shown in the heatmap. (**E**) Confocal microscopy showing the expression levels of *pCLV3::dsRED-N7* and *pWUS::ER-GFP* in the IM of a double reporter line, captured at the developmental stage of the dissected meristems used for nuclear isolation. (**F**) Heatmap plots illustrating selected marker genes from the undifferentiated cell cluster. Each gene is individually normalized subtracting its minimum value from all values and then dividing each value by its maximum value. (**G**) GO enrichment analysis from the DEGs for each cluster other than the two unknown ones. Five top GO terms sorted by fold enrichment were selected for plotting. FDR, false discovery rate.

To explore the cellular diversity within the IM of *A. thaliana*, we used an snRNA-seq approach using dissected wild-type meristems. The dissection process involved excising all flowers beyond stage 3 (*23*) and sectioning the IM around 200 to 300 μm below the apex of the meristematic tissue (Fig. 1A). Immediately following dissection, tissues were cryopreserved in liquid nitrogen for subsequent nuclear isolation (Materials and Methods). The isolated nuclei were then processed using the 10X Genomics Chromium platform and subsequently sequenced on the Illumina NovaSeq X. Three replicates were prepared and sequenced independently (fig. S1 and tables S1 and S2). Following data integration and quality control, a total of 10,025 single-nucleus transcriptomes, covering 19,491 genes, were obtained. Integrated datasets were able to reconstruct the expression profile of bulk RNA-seq obtained from the IM similarly dissected (fig. S1F). Unbiased clustering analysis of transcriptional heterogeneity led to the classification of nuclei into 18 cell type clusters (Fig. 1B; fig. S1, G and H; and tables S3 to S5; see Materials and Methods).

**Fig. 2.**
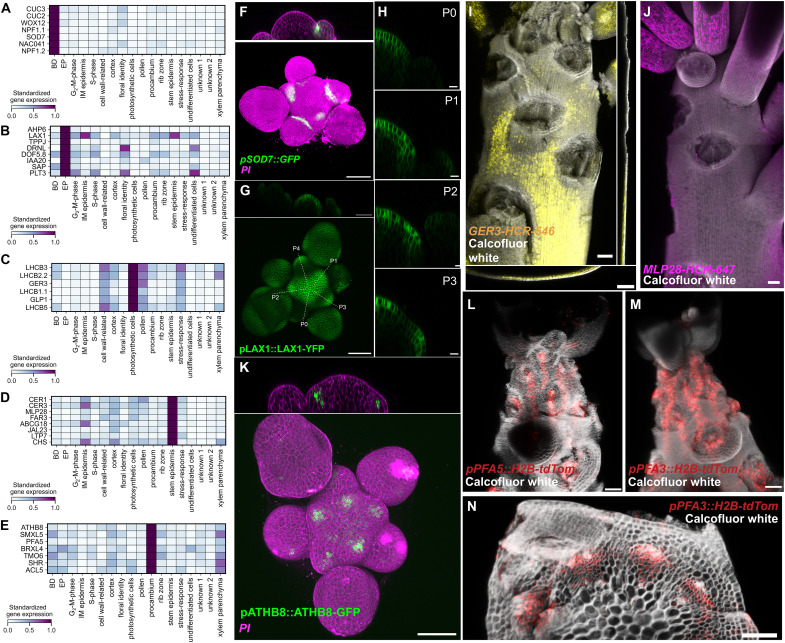
Cell clusters captured cellular identities at different differentiation states. (**A** to **E**) Heatmap plot illustrating selected marker genes from the (A) BD cluster, (B) EP cluster, (C) photosynthetic cell cluster, (**D**) stem epidermis cluster, and (E) procambium cluster. Each gene’s expression level across clusters is linearly min-max scaled. (**F**) Orthogonal projection (top) and maximum projection (bottom) of the *pSOD7::GFP* reporter line in the IM. Cell walls were stained with PI. (**G**) Orthogonal projection (top) and maximum projection (bottom) of fluorescence from the *pLAX1::LAX1-YFP* reporter in the IM. (**H**) Orthogonal primordial slices of the *pLAX1::LAX1-YFP* reporter line following the primordium formation from incipient primordia to primordium number 3. Scale bars, 5 μm. (**I**) RNA-FISH detection of the *GER3* transcript in a proximal stem section. Transverse (bottom) and orthogonal (right) projections of proximal stem. (**J**) RNA-FISH detection of the *MLP28* transcript in a proximal stem section. (**K**) Orthogonal projection (top) and maximum projection (bottom) of the pATHB8::ATHB8-GFP reporter line in the IM. Cell walls were stained with PI. (**L**) Orthogonal section of the IM and primary stem in the *pPFA5::H2B-tdTom* reporter line. Cell walls were stained with Calcofluor white. Separated channels are shown in fig. S5G. (**M**) Orthogonal section of the IM and primary stem in the *pPFA3::H2B-tdTom* reporter line. Cell walls were stained with Calcofluor white. (**N**) Transverse proximal stem section of the *pPFA3::H2B-tdTom* reporter line stained with Calcofluor white. Scale bars, 50 μm.

We used prior knowledge of marker gene expression patterns within the IM to label the observed clusters (Fig. 1C and fig. S2). The BD cluster was identified by the expression of *CUP-SHAPED COTYLEDON 2* and *3* (*CUC2* and *CUC3*) (*24*). The EP cluster was defined by genes associated with primordium formation, such as *HISTIDINE PHOSPHOTRANSFER PROTEIN 6* (*AHP6*) and *DORNRÖSCHEN-LIKE* (*DRNL*) (*10*). We distinguished between two epidermal clusters (Fig. 2D and fig. S3A). The first cluster displayed expression of genes associated with a less differentiated epidermal state, including *KANADI 1* (*KAN1*), *FIDDLEHEAD* (*FDH*), and *YABBY1* (*YAB1*) (*25*). In contrast, the second epidermal cluster exhibited strong expression of canonical cuticle and wax biosynthesis genes, including *ECERIFERUM 1* and *3* (*CER1* and *CER3*) and *CUTICULAR 1* (*CUT1*), all expressed in the stem epidermis (*26*, *27*). On the basis of the known expression of *YAB1* in the epidermis of the abaxial side of emerging primordia (*28*), specifically at the boundary between the primordia and the stem epidermis, we named these two clusters as IM epidermis and stem epidermis. Both epidermal clusters observed in our dataset shared similar expression profiles with previously reported apex-related epidermal populations (figs. S2, A to E) (*20*, *22*). The rib zone region was identified on the basis of the expression of genes previously observed in the lower region of the IM, such as *PLANT CYSTEINE OXIDASE 2* (*PCO2*) (*29*) and *SQUAMOSA PROMOTER BINDING PROTEIN-LIKE 4* (*SPL4*) (fig. S3C) (*30*). A group of cells characterized by the expression of photosynthetic genes was also observed. The expression of photosynthesis-related genes such as *LHCB3* and *LHCB2.2* has previously been identified in various stem domains (*31*). Given that this cluster may comprise cells from multiple spatial domains, we named this cluster photosynthetic cells, reflecting its functional identity. Cortex cells were defined by the expression of *JACKDAW* (*JKD*), *ASPARTYL PROTEASE* (*AED3*) (*AT1G09750*), and other genes previously observed in the root cortex (*32*). Two vascular clusters were observed. One of them was defined by the expression of procambium-active genes such as *HOMEOBOX GENE 8* (*ATHB8*) (*6*) and *ACAULIS* (*ACL5*) (*33*), defined here as procambium cluster. A second vascular cluster that we named xylem parenchyma was identified as having cells expressing vasculature-associated genes such as *GRETCHEN HAGEN 3.6* (*GH3.6*) (*19*), *FANTASTIC FOUR 1* and *3* (*FAF1* and *FAF3*) (*11*), *XTH4* (*34*), and *CLE46* (*35*) (fig. S3E). This cluster highly overlaps with a cluster identified as xylem parenchyma in a previous single-nucleus floral meristem study (fig. S2C) (*22*). We also identified a group of cells enriched in cell wall–related genes, including *PECTIN METHYLESTERASE 5* (*PME5*), *PME48*, *ARABINOGALACTAN 6* (*AGP6*), and *RAPID ALKALINIZATION FACTOR 8* (*RALF8*) (fig. S3F). Previous studies reported *PME5* expression in the SAM as a scattered pattern (*36*), and more recently, it was shown to be transiently expressed predominantly in the mature region of the IM (*37*). We therefore designated this population as the cell wall–related cluster.

We also identified a floral identity cluster expressing floral homeotic genes such as *APETALA 3* (*AP3*), *PISTILLATA* (*PI*), *AGAMOUS* (*AG*), and *SEPALLATA 1*-*3* (*SEP1*-*3*) (Fig. 1C and fig. S3B) (*38*). S phase and G_2_-M phase clusters were identified by the expression of histone-related genes and mitotic marker genes, respectively (fig. S3, D and G). A stress-related cluster, observed in vegetative apex single-cell analysis, was also detected in our dataset (fig. S3H) (*12*). A distinct cluster coexpressed *WUS*, *CLV3*, *AT3G59270*, *AT1G26680*, *LOG4*, *MCT2*, and *ZPR3* (Fig. 1, D to F), all of them expressed in the CZ and/or OC domain (*19*, *39*–*41*). Considering the expression of these genes, this cluster likely extends from the epidermis to layer 7 at the center of the IM. In addition, when compared with FACS-sorted and TRAP-based domains (*20*, *21*), this cluster highly overlapped with WUS+, CLV3+, and HDG4+ cells (fig. S2, D to F). Thus, and because we did not capture OC and CZ as isolated clusters, we named this cluster the undifferentiated cell cluster. Two unknown clusters remained unrelated to any known domain due to the lack of available reporter lines or clear Gene Ontology (GO) term enrichment. Last, a pollen cluster was defined by pollen-related genes, perhaps due to the occasional pollen grain found at the top of the meristem postdissection (fig. S3I).

Building on prior analyses of meristem-related transcriptomic profiles, we compared our inflorescence primary stem dataset with previously reported floral meristem single-nucleus transcriptomic (*22*), vegetative apex scRNA-seq (*12*), inflorescence FACS-sorted samples (*20*), and TRAP-based dataset (*21*), in terms of the number of shared DEG per cell cluster across datasets (fig. S2). We merged cluster/domain assignments in which each gene was detected in previous datasets with the marker genes identified in this study (table S4). We next asked whether there are biological functions significantly enriched in the GO categorizations of the marker genes identified in each cell type. Different biological functions were significantly enriched within each cluster with a low level of overlap (Fis. 1G, fig. S4, and table S6). Most of the observed GO terms are associated with the cell identities previously assigned on the basis of unbiased clustering and marker gene analysis. For example, the EP cluster was enriched for genes related to auxin signaling and response, the procambium cluster showed differentially enriched GO terms such as phloem or xylem histogenesis, and the BD cluster was enriched for genes related to the formation of anatomical and plant organ boundaries. We also captured immune-related genes in the vasculature xylem parenchyma cluster (Fig. 1G), consistent with previous reports that the vasculature actively contributes to immunity and systemic acquired resistance (*42*, *43*). Thus, GO term enrichment analysis accurately separated clusters by biological function, validating the differential expression analysis used to identify the marker genes in each cluster.

### Cell identities are discernible at different stages of differentiation

To assess the accuracy of unbiased clustering, we examined the expression pattern of predicted differentially expressed marker genes (Fig. 2, A to E). In the BD cluster, the gene *SUPPRESSOR OF DA1*-*1* (*SOD7/NGAL2*) has been previously characterized as repressing *CUC* expression during the initiation of axillary meristems (AMs) and it was also observed as a marker gene in our approach (*44*) (Fig. 2A). Within the EP cluster, we observed the expression of previously characterized genes such as *AHP6* and other genes associated with primordium formation (fig. S5). The spatial gene expression pattern of *AHP6* indicates that this cluster captured nuclei from the entire early primordium, without distinguishing between abaxial or adaxial sides (figs. S5, A to C). *LIKE AUXIN RESISTANT 1* (*LAX1*), an auxin influx carrier gene, has an epidermal and primordia expression pattern observed by in situ hybridization and β-glucuronidase (GUS) staining in the SAM (*45*). In our datasets, it also showed a dual expression pattern in the IM epidermis and EP clusters (Fig. 2B), which was then confirmed at the cellular level using the reporter line pLAX1::LAX1-YFP (Fig. 2G). Following the primordia formation pattern, we observed an internalization of *LAX1* expression at the beginning of primordium formation, suggesting that the action of LAX1 is to facilitate primordia initiation mainly in very early stages (P1 to P2) (Fig. 2H). The photosynthetic cell cluster was characterized by strong expression of *LHCB3* and *LHCB2.2* (Fig. 2C), two light-harvesting complex genes previously reported to exhibit a scattered expression pattern across multiple stem cell types (*31*). To further validate the identity of this cluster, we performed in situ hybridization for *GERMIN3* (GER3) (Fig. 2I), another DEG in this cell population. Using mRNA fluorescence in situ hybridization based on hybridization chain reaction (HCR-FISH) (*46*), we observed that *GER3* displayed an apical-basal gradient, with *GER3* expression becoming stronger and less scattered toward the more mature regions of the stem and in pedicels. This result indicates that the abundance of photosynthetically active cells increases as stem tissue differentiates. The expression pattern of *MLP-LIKE PROTEIN 28* (*MLP28*), a DEG enriched in the stem epidermis cluster, showed epidermal expression in the deeper stem regions and in the pedicel epidermis (Fig. 2J and fig. S5F).

The procambium cluster showed expression of genes previously described as active during vascular differentiation in roots and shoot (Fig. 2E and fig. S5D). *ATHB8* RNA, a DEG from the procambium cluster, has been observed in the procambial strand within the SAM connecting floral primordia with the primary stem (*6*). Using a translational reporter, we confirmed that the procambial pattern of ATHB8 within the SAM was associated with primordium formation following a phyllotactic pattern (Fig. 2K). We observed that some genes belonging to the procambium cluster, such as *PFA3* and *PFA5*, showed an internalized expression pattern that converged in the primary stem from developing flowers around 100 μm below the apex, as revealed by the transcriptional reporters *pPFA3::H2B-tdTom* and *pPFA5::H2B-tdTom* (Fig. 2, L and M, and fig. S5, G and H). *PFA3* showed the expected expression pattern for vascular bundles characterized during primary growth (Fig. 2N) (*47*). Together, our clustering approach successfully resolved distinct cell states and identities that captured the differentiation transition from the IM to the stem.

### GH3 family regulates shoot meristematic activity and phyllotaxis

**Fig. 3.**
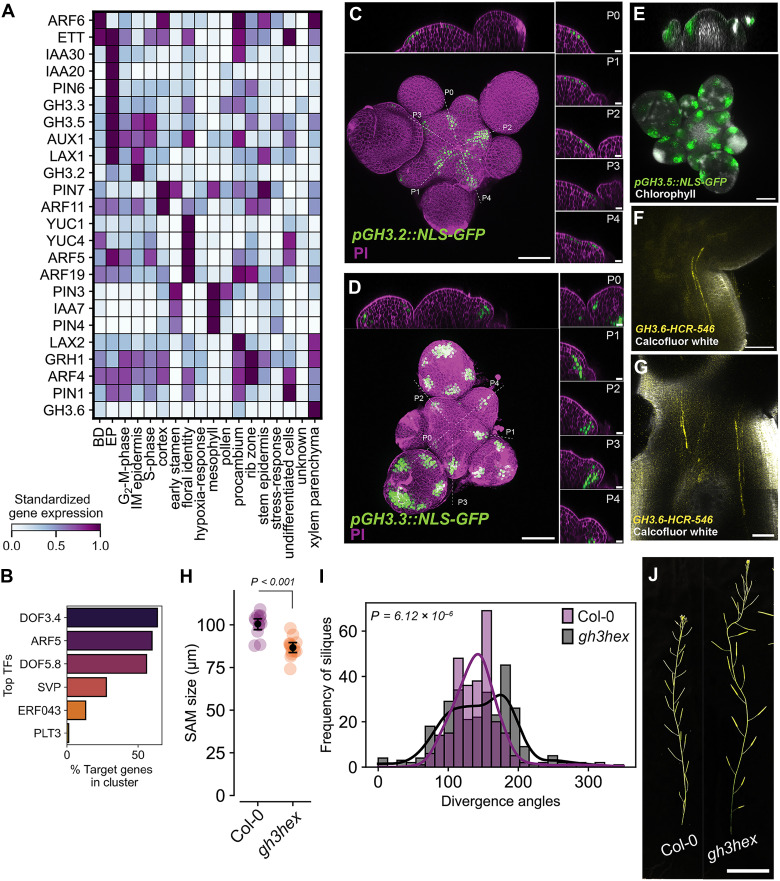
GH3 auxin conjugation family genes regulate SAM activity and affect stem torsion. (**A**) Heatmap plot illustrating normalized expression values of auxin-related marker genes. (**B**) Ranking of TF marker genes within the EP cluster based on the number of putative target genes. (**C**) Orthogonal projection (top) and maximum intensity projection (bottom) of the *pGH3.2::NLS-GFP* reporter line in the IM. The right panel shows the primordium initiation ordered from incipient primordia to primordium number 4. Scale bars, 50 μm (IM projections) and 5 μm (primordia orthogonal projection). (**D**) Orthogonal projection (top) and maximum projection (bottom) of the *pGH3.3::NLS-GFP* reporter line in the IM. The right panel shows the primordium initiation ordered from incipient primordia to primordium number 4. Scale bars, 50 μm (IM projections) and 5 μm (primordia orthogonal projection). (**E**) Orthogonal projection (top) and maximum projection (bottom) of the *pGH3.5::NLS-GFP* reporter line in the IM. Scale bar, 50 μm. (**F** and **G**) HCR detection of the *GH3.6* transcript from two different proximal stem sections. Scale bars, 50 μm. (**H**) SAM size quantification in Col-0 and *gh3hextuple* mutant lines (*gh3.1/2/3/4/5/6*). A pairwise Tukey test was conducted to quantify differences in SAM area. (**I**) Frequency of divergence angle between siliques in Col-0 and *gh3hex* mutant lines. A Kolmogorov-Smirnov test was conducted to test difference in distribution. (**J**) Principal stem section comparing Col-0 and *gh3hex* mutant lines.

We examined auxin-related genes that were differentially expressed in the identified clusters. Also supported by GO analysis (Fig. 1G), EP was the main cluster enriched in auxin-related genes (Fig. 3A). Expression of many of these genes such as *IAA30* and *IAA20* have been reported to be expressed during early primordium formation (*48*). Several auxin-related genes were highly expressed in more than one cluster (*ETT*, *IAA30*, *AUX1*, and *LAX2*), a pattern consistent with early expressed genes with a role involved in the formation of vasculature strands within newly forming primordia. Unlike the genes whose expression is associated with auxin action, the expression of genes related to other hormones such as cytokinin (CK) and gibberellic acid (GA) exhibited a more dispersed expression across different clusters (fig. S6).

Given that we identified several auxin-related genes coexpressing in the EP cluster, we investigated the GRN within this group of cells. We considered TF-target interactions that were validated by experimental evidence to establish a GRN for the DEG in this cluster (Materials and Methods). AUXIN RESPONSE FACTOR 5/MONOPTEROS (ARF5/MP), DOF3.4, and DOF5.8 were identified as the most connected TFs within this cluster, validating the GRN approach to obtain potential master regulators for a single cellular identity (Fig. 3B and table S7). ARF5/MP has been previously reported as a master regulator of primordium formation in response to auxin and DNA-binding with One Finger (DOF) TFs have been postulated to act downstream of ARF5/MP (*49*). DOF5.8 and DOF3.4 have been suggested to act redundantly based on sequence similarity (*49*). Although GRN analysis indicated three potential master regulators during primordia formation, we observed a specific role of ARF5/MP in auxin-related functions due to its exclusive interaction with DNA binding sites associated to INDOLE-3-ACETIC ACID INDUCIBLE 30 (IAA30) and IAA20 (fig. S7A). Our analysis also highlights the overlapping role of DOFs and ARF5/MP as master regulators of primordium formation through the regulation of a similar set of genes such GH3.2, ETT, and AUX1.

In addition, we detected expression of auxin-related genes with unknown functional roles in shoot meristematic tissue, including the auxin amido synthetases *GH3.2*, *GH3.3*, *GH3.5*, and *GH3.6*. We validated the expression pattern for all four genes (Fig. 3, C to G). As suggested by the transcriptomic analysis, *GH3.2* was predominantly expressed in the IM epidermis, whereas *GH3.3* and *GH3.5* expression was associated with developing primordia. In contrast, as previously observed (*19*), HCR-FISH confirmed a vascular expression pattern for *GH3.6* (Fig. 3, F and G). Together, at least three of the four differentially expressed GH3 genes detected in the IM showed expression related to the developing primordia. Because of the reported functional redundancy, we assessed the role of the GH3 family in meristematic activity using the sextuple mutant line *gh3.1/2/3/4/5/6* (*gh3hex*) (*50*). Neither *GH3.1* nor *GH3.4* transcripts were detected in our single-nucleus transcriptomic dataset. All of the GH3 proteins share the similar enzymatic activity of conjugating auxin with aspartate: *gh3hex* mutants lost the ability to form IAA-Asp but not IAA-Glu (*51*). In the *gh3hex* mutant line, we observed a meristem-related phenotype, including smaller meristems compared to wild type and extended meristem activity that supported prolonged production of flowers (Fig. 3H and fig. S7, B to E). In addition, the phyllotactic pattern was shifted in the sextuple mutant lines compared to wild type with higher frequency of 180° and 90° divergence angles between siliques. As auxin dynamics are known to influence differential tissue twisting during tissue growth (*52*–*54*), disruption of GH3-mediated auxin conjugation may alter stem torsion, giving rise to the observed phyllotactic pattern (Fig. 3, I and J) (*55*).

### Stem cortex diverges from vasculature during primordium formation in the SAM

**Fig. 4.**
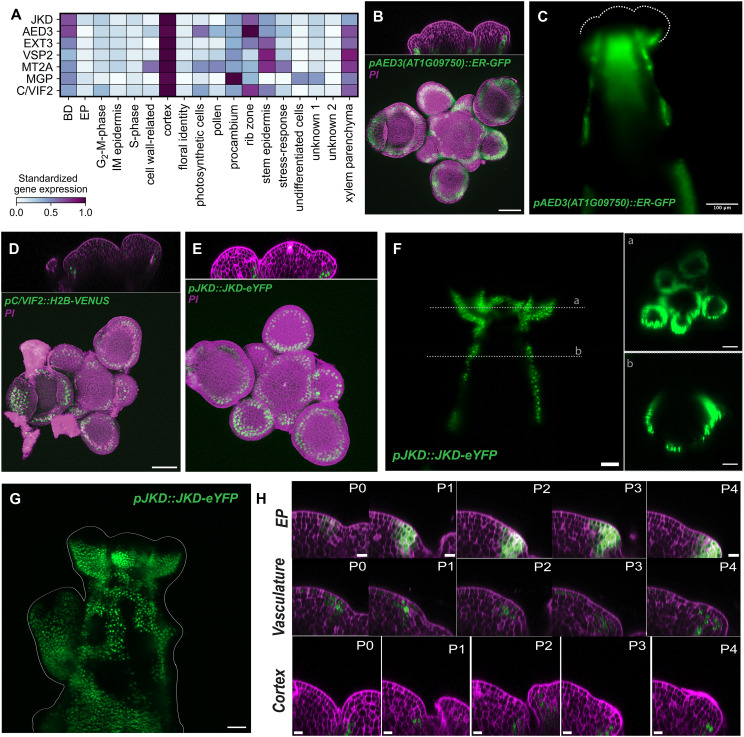
Cortex and vasculature identities diverge during primordium formation within the IM. (**A**) Heatmap plot illustrating selected cortex-related marker genes with normalized gene expression by gene. (**B**) Maximum projection of IM from the *pAED3* (*pAT1G09750*)*::ER-GFP* reporter line stained with PI. (**C**) Orthogonal projection of the light sheet image of the *pAED3* (*pAT1G09750*)*::ER-GFP GFP* reporter line. (**D**) Orthogonal and maximum projection of IM from the *pC/VIF::H2B-VENUS* reporter line stained with PI. (**E**) Orthogonal and maximum projection of IM from the pJKD::JKD-eYFP reporter line stained with PI. (**F**) Orthogonal slice of the light sheet image of the pJKD::JKD-eYFP reporter line with two selected transverse sections observed in the right panel. (**G**) Maximum projection of IM from the pJKD::JKD-eYFP reporter line. Inflorescence epidermal contour was labeled using autofluorescence signal. (**H**) Primordium formation in the *pAHP6::ER-GFP* reporter line (top; same images as in fig. S5B, shown with a different crop), *pATHB8::ATHB8-GFP* reporter line (middle; from Fig. 2K), and pJKD::JKD-eYFP reporter line (bottom; from E) following the phyllotactic pattern. Scale bars for primordium initiation, 5 μm. Scale bars, 50 μm.

Secondary growth analysis and stained stem sections have shown the presence of the cortex in stem tissue, positioned between the epidermis and vascular bundles (*31*). However, the precise timing and molecular mechanism governing cortex formation in stems is unstudied. By unbiased clustering, we identified a cluster enriched in the expression of genes that have previously been associated with the root cortex such as *JKD*, *AED3*, and *C/VIF2* (Fig. 4A) (*32*). Through analysis of reporter lines pJKD::JKD-YFP, *pAED3::ER-GFP*, and *pC/VIF2::H2B-VENUS*, we observed an expression pattern localized to layer 2 (L2) within developing primordia. Orthogonal views of the SAM using reporter lines revealed cortex-related gene expression during primordium formation (Fig. 4, B to G). Because L1 and L2 layers in the SAM are characterized by anticlinal division, cortex identity may be generated from L2 cell lineages that originate in the CLV3 domain. To obtain observations farther below the meristem in the primary stem, we assessed *AED3* and *JKD* expression using light sheet microscopy. We observed that the *AED3* promoter is active in a path from early developing primordia downward through the stem (Fig. 4, B and C). The use of light sheet microscopy also allowed us to capture the dual expression pattern of *AED3* in the cortex and rib zone, as predicted by our clustering analysis (Fig. 4A). JKD also showed a similar expression pattern with cells expressing *JKD* during EP toward the primary stem (Fig. 4F). We observed *JKD* expression in pedicel and part of the mature flower connecting flowers to the IM (Fig. 4, F and G). Gaps in expression in the stem were observed, corresponding to developing flowers we had removed during the dissection process.

Although *JKD* expression has been previously observed in the SAM (*56*), the identification of a group of marker genes with a similar expression pattern confirms a previously uncharacterized domain of cell identity within the SAM that will subsequently generate the cortex in the pedicel, flowers, and stem. Following primordium formation in the *JKD* reporter line, we observed that the expression of this cortex marker gene coincides with morphological changes during primordium formation on the abaxial side of the new organ (Fig. 4H). Both EP (fig. S5, A and B) and procambium marker genes (Fig. 2K) were observed during primordium formation, coexpressing with cortex-related genes within this spatial domain. Although vasculature stripes form within the middle of the primordia, cortex identity appears in L2. In roots, cortex specification is governed by the well-characterized SHR-SCR regulatory network (*57*). Whether stem cortex relies on a similar mechanism has also been explored (*56*). Consistent with this, our dataset captured *SHR* expression in the procambium cluster (Fig. 2E), adjacent to the expression in the primordia. We further expanded this network by observing that *MAGPIE* (*MGP*), which acts redundantly with JKD in roots (*58*), displays a dual expression pattern in the shoot meristem cortex and procambium clusters (Fig. 4A). Together, our single-nucleus transcriptomic analysis and reporter validations identify transcriptional states consistent with a bifurcation between cortex- and vascular-associated gene expression patterns within developing primordia. These findings establish a testable framework for investigating coordinated cortex and vascular specification during stem development.

### S phase is enriched in floral-related domains

Although many molecular players underlying IM cell cycle dynamics have been characterized (*59*), a comprehensive overall transcriptional description of cell cycle–related gene expression in the SAM remains to be created. We investigated the cell cycle dynamics of the IM through trajectory inference (TI) analysis using the two identified cell cycle–related clusters: S phase and G_2_-M phase.

**Fig. 5.**
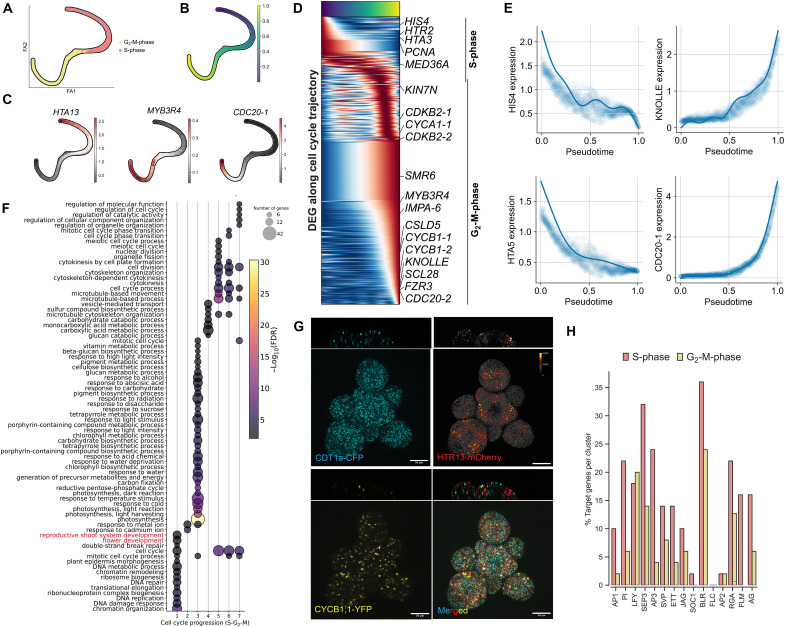
Diverse cell cycle transcriptional profiles in IM cell types. (**A**) Force-directed graph layout of clusters associated to the cell cycle (S phase and G_2_-M phase). Clusters are represented by different colors. (**B**) Pseudotime estimation of cell cycle progression. Color bar represents pseudotime. (**C**) HTA8, HTR13, and CDC20-1 expression along the cell cycle trajectory. (**D**) Heatmap displaying clustering of DEGs based on the maximum expression profile along the cell cycle trajectory. (**E**) Expression trends of selected DEGs along the cell cycle progression. (**F**) GO analysis of DEGs along the cell cycle trajectory. Genes were ordered by peak expression and sampled in group of 100 genes. (**G**) Confocal image of IM from PlaCCI lines showing CDT1a (G_1_ phase), HTR13 (S phase), and CYCB1;1 (G_2_-M phase) reporters. The S phase marker line is shown as the heatmap of fluorescence intensity. Scale bars, 50 μm. (**H**) Percentage of target genes per cluster for floral homeotic genes based on ChIP-seq datasets.

We integrated diffusion maps, multiscale diffusion space, and cell-cell neighbor graphs to construct force-directed trajectories (Materials and Methods) (Fig. 5A). On the basis of a structure of a reconstructed trajectory tree, we defined an S phase edge as the root for pseudotime, representing the earliest point in the cell cycle progression (Fig. 5B). Gene expression of histone-related proteins and M phase genes confirmed that dimensionality reduction captured overall cell cycle dynamics (Fig. 5C). Cubic spline regression was applied to model gene expression along pseudotime, generating smooth fitted expression profiles that identified 2003 DEGs with a *P* value of <0.01 (table S8). Consistent with known cell cycle dynamics, histone-related genes exhibited an early expression peak in the reconstructed cell cycle trajectory, succeeded by the expression of G_2_-associated genes such as *CYCA1;1* and *CDKB2;1* (*60*). Last, genes associated with M phase, including *KNOLLE* (*KN*) and *CELL DIVISION CYCLE 20*-*1* and *20*-*2* (*CDC20*-*1* and *CDC20*-*2*), were expressed in the later region of the trajectory (Fig. 5, D and E, and fig. S8A) (*59*).

Then, we asked whether the conducted trajectory analysis could capture cell cycle–specific biological dynamics. We performed GO enrichment analysis across pseudotime-ordered DEGs, grouped by their peak expression along the trajectory (Fig. 5F). This approach allowed us to associate gene biological terms with specific stages of the cell cycle. As expected, early pseudotime was enriched for chromatin organization and DNA replication terms, whereas later pseudotime included terms related to cytokinesis and microtubule-based processes (table S9). Unexpectedly, S phase–enriched genes were also associated with GO terms related to regulation of the reproductive shoot system and flower development, which is also supported by highly expressed histone-related genes in the floral identity cluster (fig. S3G).

To test whether S phase is related to flower development, we used the PlaCCI cell cycle reporter line (*61*). We observed that G_2_-M phase markers showed a uniformly speckled pattern as has previously been reported for a CYCB1;1 translational reporter and other G_2_-M–related genes (*59*) (Fig. 5G). On the contrary, the histone H3.1 S phase reporter line *pHTR13::HTR13-mCherry* showed a peak of expression in particular domains such as EP and central meristematic floral domains, coinciding with the expression pattern of some floral homeotic proteins such as the ring pattern in the central floral meristem of *AP3* and EP expression pattern of *AP1* (Fig. 5G and fig. S8, B and C) (*62*–*64*). Thus, although G_2_-M phase–related genes showed a uniformly speckled expression pattern in the meristem, the expression pattern of some S phase–related genes, although expressed in a speckled pattern, are preferentially distributed in domains associated with the coexpression of floral homeotic genes. Length of the S-G_2_-M phase has been shown to be flexible depending on the primordium stage (*65*), supporting the heterogeneous expression pattern that we observed for the S phase reporter line.

We investigated whether floral homeotic TFs bind to genomic regions of S phase–expressed mitotic genes. Genome-wide TF binding datasets for several floral homeotic genes have been previously reported (*66*). Floral regulator binding sites were found to be highly enriched near S phase–related genes compared to G_2_-M phase–related genes (Fig. 5H), suggesting that floral homeotic TFs may potentially contribute to the regulation of S phase–related genes in the IM. For instance, 32% of the DEGs in the S phase cluster are direct targets of AP3, whereas only 14% of the G_2_-M phase genes are targets of this TF. We observed similar behaviors for most of the floral homeotic TFs assessed, supported by the direct binding of SEP3 to histone-related genes such as HTR8, HTA5, HTA2, and HTA13 or by the direct binding of AG to the promoter of HTA2 or HTR2 (table S10).

### Multiple differentiation events connecting shoot stem cells and the primary stem

**Fig. 6.**
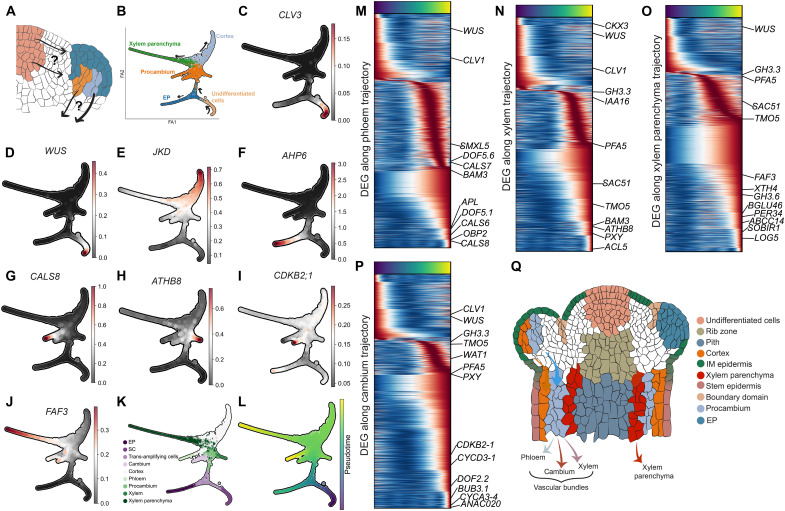
Dynamic transcriptional differentiation of inner cell identities within the IM and primary stem. (**A**) Scheme representing the differentiation trajectories between undifferentiated cell cluster (pink) and downstream cell identities. (**B**) Force-directed graph layout of clusters associated with inner cell layers such as the EP, undifferentiated cells, procambium, xylem parenchyma, and cortex. Clusters are represented by different colors. (**C**) *CLV3* expression within the force-directed graph layout. (**D**) *WUS* expression within the force-directed graph layout. (**E** to **J**) *JKD*, *AHP6*, *CALS8*, *ATHB8*, *CDKB2;1*, and *FAF3* respective expression within the force-directed graph layout. (**K**) Force-directed graph layout of identified cell types according to branch-specific gene expression. Domains were defined by milestones according to bifurcation points. Boundaries between domains are schematic and not intended to represent precise biological limits. (**L**) Pseudotime analysis using OC/CZ-like edge with WUS+ and CLV3+ cells as the initial point. (**M**) Heatmap of DEGs along the phloem trajectory. (**N**) Heatmap of DEGs along the xylem trajectory. (**O**) Heatmap of DEGs along the xylem parenchyma trajectory. (**P**) Heatmap of DEGs along the cambium trajectory. (**Q**) Schematic illustration of selected cell identities in the primary stem and IM. Procambial strands arise in developing primordia and give rise to vascular bundles, which subsequently differentiate into the phloem, cambium, and xylem within the primary stem. The exact locations of cell identities in the primary stem are schematically represented rather than accurately portrayed.

We then aimed to trace the trajectories of differentiation from stem cells to the various cell types observed during primordia formation such as the cortex, vasculature, and EP (Fig. 6A). On the basis of validated and published expression patterns (Figs. 2 and 3), we performed TI analysis between the undifferentiated cell cluster and other identities that we validated to start the differentiation process within inner cells of the IM such as the procambium, xylem parenchyma, cortex, and EP (Fig. 6B). Similar to our analysis for cell cycle trajectory analysis, we used diffusion maps, multiscale diffusion space, and cell-cell neighbor graphs to construct a force-directed trajectory (Materials and Methods). The number of eigenvalues used to reduce dimensionality were selected by increasing until biologically meaningful branches were observed from combined clusters (fig. S9). Notably, reducing dimensionality positioned WUS+ and CLV3+ cells at the tip of the undifferentiated cell cluster branch (Fig. 6, C and D). In addition, previously identified and validated genes from different cell types were expressed in specific branches of the trajectory, such as *JKD* expressed in the cortex branch and *AHP6* expressed in the EP branch (Fig. 6, E and F).

TI analysis separated the procambium cluster into three different branches with distinct transcriptional profiles. One branch showed the expression of genes whose functions are associated with phloem development such as *CALLOSE SYNTHASE 8* (*CALS8*) (Fig. 6G) (*67*), another branch showed the expression of genes related to development of xylem such as *ATHB8* (Fig. 6H), and a third branch showed expression of genes whose transcription is associated with cell division–related genes, as expected for cambium cells (Fig. 6I) (*68*). The xylem parenchyma cluster did not split into branches, and it was defined by the expression of vascular genes such as *GH3.6*, *FAF1*, and *FAF3*, which have an internalized expression pattern not related to phyllotaxis (Fig. 6J) (*11*). Thus, trajectory analysis enabled us to infer transcriptional trajectories consistent with differentiation toward cell types observed in the primary stem such as the cortex, xylem, phloem, cambium, and xylem parenchyma (Fig. 6K).

By defining WUS+ and CLV3+ edge as the pseudotime root (Fig. 6L), we modeled gene expression along the different branch trajectories and applied a cubic spline regression independently to each differentiation branch to capture expression trends (Materials and Methods). By using a *P* value of <0.01, we identified 2401 DEGs during phloem differentiation (Fig. 6M), 1908 DEGs along xylem differentiation (Fig. 6N), 3006 DEGs for xylem parenchyma (Fig. 6O), 1844 DEGs along the cambium trajectory (Fig. 6P), 940 DEGs along EP differentiation (fig. S10, A and B), and 3409 DEGs in cortex differentiation (fig. S11A and tables S11 to S16). Concomitant with our microscopy observations (Fig. 4H), the first bifurcation point in our trajectory analysis corresponded to the EP cluster. This enabled us to examine transcriptional dynamics consistent with transit-amplifying cells in the PZ, located between the WUS/CLV3 stem cell domain and the developing primordia (fig. S10A). Along the pseudotime trajectory, we captured the coordinated decrease in expression of the stem cell markers *CLV3* and *WUS*, whereas expression of genes within EP such as *AHP6* and *DRNL* gradually increased (fig. S10, B and C). To capture the dynamics of biological processes, DEGs were ordered by peak expression and binned along pseudotime, and GO enrichment analysis was performed for each window. This approach confirmed that stem cell–related processes were enriched at the beginning of the trajectory (WUS/CLV3+ cells), whereas auxin-related processes appeared specifically at the end of the trajectory (EP). In addition, it revealed previously unappreciated transcriptional dynamics, including transient enrichment of hypoxia- and translation-related genes during the transition of these transit-amplifying cells.

Within the phloem trajectory we observed the expression of genes characteristic of differentiated phloem such as *CALS8* (*69*), *OBP2* (*70*), and *PEAR2/DOF5.1* (*70*) (Fig. 6M). The xylem-related trajectory showed *TMO5*, *ATHB8*, and *ACL5* as DEGs (Fig. 6N). The xylem parenchyma trajectory exhibited a similar expression pattern with early-expressed interfascicular RNAs such as the transcripts of *FAF1* and *FAF3* and with late-expressed interfascicular genes such as *BETA GLUCOSIDASE 46* (*BGLU46*), the product of which participates in lignification, a process observed in xylem differentiation (Fig. 6O) (*71*). The cambium trajectory also showed the expression of *PXY* and cell cycle–related genes such as *CYCA3;4* (Fig. 6P and fig. S11B). Thus, we were able to capture the transcriptional landscape of differentiation processes for a variety of cell identities defined between the SAM and the primary stem.

## DISCUSSION

Leveraging single-nucleus transcriptomics, we provide a quantitative map of cell identities and differentiation processes occurring within the IM and emerging primary stem in *A. thaliana* (Fig. 6Q). Cross-dataset comparisons clarify both convergence with, and added value of, our study relative to earlier work. As in FACS-sorted approaches, our single-nucleus dataset robustly captures the central undifferentiated region of the SAM, enriched for *WUS*, *CLV3*, and *HDG4* expression, and provides a clear single-cell/nucleus transcriptomic dataset in which this domain appears as a well-defined isolated cell population. Unbiased single-nucleus profiling also revealed differentiation progression and transitional states that are not readily resolved in bulk-sorted populations. Cluster-to-cluster comparison with previously published datasets further underscored the importance of fine tissue dissection for isolating rare cell types such as EP, BD, and undifferentiated cells using single-cell transcriptomic approaches (fig. S2). Although the presence of a stress-related cluster in our dataset, as well as in vegetative apex STM+ cells, may reflect artifacts introduced during cell or nuclei isolation, GO analysis of the DEGs in this cluster is consistent with the intrinsically hypoxic microenvironment of the SAM (*29*), suggesting at least a partial signature of biological context. Alternatively, increasing the resolution by adding more nuclei to our analysis and improving the nuclear extraction from frozen tissue may help us to increase the variety of cell identities observed in the SAM.

We expanded the repertoire of auxin-related genes within the IM such as the influx carrier *LAX1*, *GH3.2*, and *GH3.3*. The potential role of auxin inactivation by its conjugation into IAA-Asp by GH3.2, GH3.3, and GH3.5 may add a new layer of regulation to the morphogenesis of developing primordia. The absence of multiple GH3 enzymes in the *gh3hex* mutant is expected to result in elevated or prolonged levels of active auxin in developing primordia, which may explain the extended meristem activity compared to wild-type plants (fig. S7E). Because additional GH3 members are expressed in other domains such as GH3.6 in xylem parenchyma, further analysis with the triple mutant *gh3.2/3/5* could help us understand the specific role of EP-expressed GH3 members. The overlapping expression of DR5 and several GH3 members in the shoot meristem, and during lateral root development (*50*), suggests that DR5 expression could also indicate auxin inactivation such as by conjugation and thereby a negative feedback rather than purely downstream auxin signaling outputs.

Validation with several reporter lines revealed that the previously unidentified cortex cell identity is generated in the L2/L3 during primordium formation (Fig. 4). *JKD* expression has previously been observed within this meristematic domain (*56*), where components of the SHR-SCR network were shown to be active in the SAM. By expanding the repertoire of genes expressed in this domain, we now show that SHR and JKD exhibit expression patterns associated with the vasculature and cortex initial cell type differentiation, respectively. This shoot-rewired SHR-SCR regulatory network suggest a role in vasculature-cortex specification, in contrast to the cortex-endodermis specification described in roots (*57*). Together, our results extend SHR-SCR models proposed for the SAM by revealing a broader cortex-associated transcriptional landscape. Although *jkd-4* mutants primarily show SAM size phenotypes (*56*), functional redundancy (for example, with MGP) may obscure cortex-specific phenotypes, motivating higher-order mutant analyses.

We described highly enrichment of S phase target genes for floral homeotic genes, supported by DNA binding evidence from previously published flower chromatin immunoprecipitation sequencing (ChIP-seq) datasets (Fig. 5H) (*66*). S phase regulation could influence morphogenesis and cell fate decisions in plants, as seen in various aspects of animal development such as development of the *Drosophila* central nervous system (*72*) or regulation of cell fate switches during erythroid differentiation (*73*). Understanding whether the S phase–enriched regions in floral domains are formed due to shorter M phase or prolonged S phases could shed light on the role of S phase in shaping plant morphology.

Integrating clonal lineage tracing with spatial transcriptomics will be essential to determine which cell identities are chiefly determined by lineage history or spatial position. Although the exceptionally small size represents a major technical challenge for spatial omics in the *Arabidopsis* SAM, recently validated methods such as multiplex smRNA-FISH (*74*), or MERFISH (*75*), could be strategically leveraged in combination with our dataset to enable rational probe design, precise spatial boundary definition between clusters, and lineage mapping. Together, by elucidating the differentiation-associated transcriptional profiles, our study characterizes multiple cell fate specification processes in wild-type IMs, thereby paving the way for future investigations into the dynamic nature of cell identity and development in plants.

## MATERIALS AND METHODS

### Plant material and growth conditions

Seeds were sterilized using 70% (v/v) alcohol for 7 min and subsequently plated on 1/2 Murashige and Skoog basal medium (MS) (pH 5.7). Seeds were stratified in the plates for 48 hours at 4°C in the dark, and plates were then positioned horizontally in a growth chamber set to 22°C with a 16-hour light/8-hour dark photoperiod. Ten-day-old seedlings were transplanted into 9-cm pots filled with Levington F2 compost and placed in a growth chamber set to 22°C with a 16-hour light/8-hour dark photoperiod.

### Bulk RNA-seq analysis

Samples were collected in 1.5 ml of RNase-free Eppendorf tubes submerged in liquid nitrogen. For each sample, three replicates were collected by pooling 30 to 40 dissected meristems per replicate. The tissue samples were homogenized using a micropestle, and total RNA was isolated using the RNeasy Plant Mini Kit (QIAGEN, 74904) following the manufacturer’s instructions. The RNA quality was evaluated with an Agilent 2200 TapeStation by Novogene Ltd. (UK). The libraries were prepared with polyA enrichment and sequenced at a depth of 30 million (9-Gb raw reads) with an Illumina HiSeq-PE150 by Novogene Ltd. (UK). Genome annotation against the TAIR10 reference genome (www.arabidopsis.org) was performed using hisat2 (https://github.com/DaehwanKimLab/hisat2). After alignment, raw counts were normalized using pyDESeq2

### Phyllotactic pattern measurements

To determine the phyllotactic pattern along the stem, we measured the angular position of the 13 to 20 oldest siliques from fully grown stem of 3 weeks after bolting plants. The stems were positioned with the apex facing downward, secured and extended in the central part of the device previously described (*76*). Because the phyllotactic orientation can be either clockwise or anticlockwise, the phyllotaxy orientation was standardized by the direction that resulted in the smallest average divergence angle.

### Nuclear isolation and 10X snRNA-seq library preparation

A total of 450 dissected Col-0 SAMs from three biological replicates (100, 50, and 300 meristems, respectively) were used for the snRNA-seq approach. Meristems were dissected shortly after bolting (with a stem length of 1 to 3 cm; flowers beyond stage 3 were removed). The stem was cut just below the SAM (∼200 to 300 μm; Fig. 1A), and tissues were collected and frozen in an Eppendorf tube using liquid nitrogen. Each sample was grinded using a micropestle, and then 400 μl of Honda buffer [2.5% Ficoll 400, 5% Dextran T40, 0.4 M sucrose, 10 mM MgCl_2_, 1 μM dithiothreitol, 0.5% Triton X-100, 1 tablet/50 ml of cOmplete Protease Inhibitor Cocktail (Roche), RiboLock (0.4 U/μl; Thermo Fisher Scientific), and 20 mM Tris-HCl (pH 7.4)] was added. Subsequently, 500 μl of Honda buffer was added to the tubes, and the samples were poured into small petri dishes. A 4.5-ml Honda buffer was added, and the samples were agitated at 80 rpm for 10 min. The suspension was filtered through a 70-μm strainer (Sigma-Aldrich) and then filtered again through a 40-μm strainer (Sigma-Aldrich). The filtered solution was centrifuged at 1500*g* for 6 min at 4°C. The pellet was resuspended carefully in 300 μl of landing buffer [1X phosphate-buffered saline (PBS) with 4% bovine serum albumin (BSA), Ambion RNase Inhibitor (2 U/μl), and SUPERase·In RNase Inhibitor (1 U/μl)]. Nuclei were stained with propidium iodide (PI) and sorted by gating on the PI peak intensity using a BD Bioscience Influx cell sorter with a 100-μm nozzle at a 20-psi pressure. Sorting process required about 20 to 30 min. The PI signal was detected with a 75-mW 561-nm laser using a 670/30 band-pass filter. Nuclei were collected into a maximum of 43-μl landing buffer. Replicates 1 and 3 required an additional concentration step, where nuclei were centrifugated at 1500*g* and 6 min at 4°C followed by pellet resuspension in 43 μl of landing buffer. An estimated number of 5000, 12,000, and 20,000 nuclei for each replicate, respectively, were loaded onto a 10X Chromium chip for library preparation using Chromium Single Cell 3′ Reagent Kits v3 according to the manufacturer’s instructions. The entire process, from nuclear isolation to chip loading, required ∼1 hour and 30 min. Each library was sequenced independently using the Illumina NovaSeq X Sequencing System.

### snRNA-seq data analysis

Fastq files were processed with Cell Ranger v3.1.0, using default parameter values and reads were aligned to the *A. thaliana* TAIR10 reference genome (https://arabidopsis.org/download/list?dir=Genes%2FTAIR10_genome_release). Ambient mRNA (nucleus-free RNA molecules in the cell suspension) was removed using the CellBender software package with default parameters (https://github.com/broadinstitute/CellBender). Filtered genes-by-cell matrices for each batch were concatenated and analyzed using the Scanpy package. To identify doublets, we applied Scrublet with parameters: expected_doublet_rate = 0.05, min_counts = 2, min_cells = 3, min_gene_variability_pct = 85, and n_prin_comps = 30. Cells whose transcriptome contained more than 10% mitochondrial genes were removed. In addition, cells with a number of detected genes lower or greater than five times the median were excluded (*77*). Gene transcripts present in fewer than 10 cells and organellar genes were excluded from further analysis. Genes related to the nuclear isolation process were removed on the basis of a linear regression analysis between bulk RNA-seq and snRNA-seq transcriptional profiles. By calculating residual values (observed minus expected expression), we identified and removed the top 58 genes with extreme deviations (cutoff of |5|), likely resulting from the isolation process. We normalized the datasets for cell library size to 10,000 counts without excluding highly expressed genes, log transformed, and selected 1000 top highly variable genes (HVGs) using the “seurat_v3” algorithm for the dispersion-based method. Inspection of the cumulative gene contribution to total counts showed that the top 20 genes disproportionately accounted for the library size. These genes were removed only for clustering analysis. We regressed out the total counts and percentage of mitochondrial gene per cell as a source of variation, and gene counts were scaled to mean zero and unit variance. For data visualization, we selected 50 principal components and constructed a k-nearest neighbor (kNN) graph with 50 neighbors. Replicates were integrated with Harmony, and we performed a uniform manifold approximation and projection (UMAP) using Leiden community detection with a 1.3 resolution (table S2). A Wilcoxon rank sum test with tie correction and a Benjamini-Hochberg *P* value correction method was conducted to identify marker gene transcripts [*P* value_adj < 0.01, log_2_FC (fold change) > 1, and pct_nz_group > 0.05].

#### Gene ontology

The g:Profiler package was used throughout the manuscript. Fold enrichment was calculated as the ratio between the observed proportion based on the background gene set. Enriched terms were filtered using the following criteria: adjusted *P* value < 0.01, intersection size > 5, and term size < 1000.

#### EP gene regulatory network

Because marker genes within a cluster share gene expression pattern within the same group of cells, we cross-referenced the DEGs in EP with available TF binding information from DNA affinity purification sequencing (DAP-seq) (table S7) (*78*).

#### Comparison with apex-related transcriptional profiles

DEGs identified from the vegetative apex (*12*), *ap1*-*1 cal-1* floral meristems (*22*), *ap1*-*1 cal-1* FACS-sorted domain (*19*, *20*), and TRAP-based 7 DAG seedlings shoots (*21*) were compared with the gene expression levels in our snRNA transcriptomic dataset. Expression levels of shared genes between datasets were analyzed across clusters defined in previous studies and those identified using our approach. For both scRNA-seq and snRNA-seq data, we used the DEGs as defined by the original authors. In the case of FACS-sorted cells, only genes that were defined as DEGs between in the pairwise comparisons were selected. Then, the domain with highest expression levels was assigned to each gene. For Sankey plots, regulated genes per domain in our dataset were mapped to domains defined in the previous analysis. Because each dataset used different cell type labels, we standardized the comparison by creating meta-clusters. For example, the “Vasculature” meta-cluster included DEGs from the xylem, xylem parenchyma, procambium, or phloem in each dataset (fig. S2).

### Trajectory analysis

To infer developmental trajectories in clusters of interest, data were extracted from the integrated Scanpy objects and analyzed using the Palantir (*79*) and scFates packages (*80*, *81*). To linearly reduce the dimensionality of clusters, we first obtained the diffusion component using the Palantir “run_diffusion_maps” function from the 50 principal components obtained for the cluster of interest. Subsequently, a multiscale data matrix was generated using a defined number of eigenvectors from the diffusion map using “determine_multiscale_function” from Palantir. Three and nine eigenvectors were used for cell cycle and inner cell types trajectories, respectively. Gene expression values were imputed using MAGIC for visualization and smoothing of gene dynamics along pseudotime (*82*). Using the obtained multiscale data matrix, we computed the neighborhood graph for each cell using the scanpy.pp.neighbors function with 50 nearest neighbors. Then, a force-directed graph using the first two principal components was used to infer trajectories with default parameters (*83*)

Along the obtained trajectory, a principal tree was inferred using scfates.tree function (method = “simple ppt algorithm”). Each cell is assigned a probability of belonging to different nodes in the tree. Each node of the tree contains values for all cells, with each cell having an assignment strength to a node between 0 and 1, where 1 indicates the closest proximity to the node. Root cells were selected depending on the trajectory topology (S phase or WUS+/CLV3+ cells for cell cycle and inner cells trajectories, respectively). Pseudotime values were generated as a distance on the tree from the selected roots and projected to cells. For the cell cycle trajectory, pseudotime values were rank transformed using “scipy.stats.rankdata” with the “average” method, which assigns to each value its average rank.

To identify the set of genes significantly associated with the trajectory, we used the “scfates.tl.test association” function from the scFates package. This function models feature expression as a function of pseudotime for each branch independently using a cubic spline repression (*g_i_* ∼ *t_i_*), where *g_i_* is the expression of gene *i* and *t_i_* is the pseudotime of cell *i*. This tree-dependent model is then compared to an unconstrained model (*g_i_* ∼ 1), where gene expression is assumed to be independent of pseudotime, using an *F*-test to evaluate the significance of the association. Genes with significant associations were then fitted using a generalized additive model (GAM) to derive smoothed expression trends over pseudotime (scFates.tl.fit). These fitted trends were visualized as a heatmap (‘scfates.pl.trends’), ordered by the maximum fitted value of each feature along the pseudotime trajectory (tables S11 to S16).

#### Cell cycle GO analysis

GO enrichment analysis was conducted on DEGs along the cell cycle trajectory. A hypergeometric test was applied in four sliding window, ordered by peak expression from the beginning of the S phase to the end of the G_2_-M phase (table S9).

### Microscopy analysis of fluorescent markers

#### Confocal microscopy

Shortly after bolting (stem length ∼1 cm), the shoot apices were cut and SAMs were dissected, removing most of the flowers as it was previously described (*84*). The apices were transferred to a small petri dish containing MS medium (pH 5.7). Once in the plates, the SAMs were submerged in water and the remaining flowers were removed. To show cell boundaries, each SAM cell walls were stained with 10 μl of PI solution (1 mg/ml, w/v) for 10 min. Cross sections of inflorescence stem were generated using razor blades and stained with Calcofluor white. Confocal *z*-stacks of SAMs were acquired with a Zeiss LSM880 Confocal microscope using a 25x, 0.95–numerical aperture (NA) water dipping objective. Laser excitations were 488 nm [green fluorescent protein (GFP) and yellow fluorescent protein (YFP)] and 561 nm [red fluorescent protein (RFP) and PI]. Fluorescence intensity was measured in Fiji ImageJ (https://imagej.net).

#### Quantification of SAM size

The size of the SAM was measured by determining the minimum circumference that fits the maximum projection of the dissected meristem. The circumferences were chosen to enclose the BD of three separated primordia in the IM. The diameter of the meristem was then calculated from the area enclosed by this circumference.

#### Light sheet microscopy

Light sheet imaging was performed on a custom-built light sheet microscope (*85*). Excitation and emission water immersion objectives are arranged on a horizontal plane, perpendicular to each other so that their focal volumes coincide. The sample is mounted upside down in a pipette tip filled with agarose, with the part to be imaged protruding at the bottom. The excitation objectives (Nikon 10x CFI Plan Fluorite Objective, 0.3 NA) and the two emission objectives (Olympus 20X XLUMPLFLN 20x, 1.0 NA) are facing each other, respectively. The light sheet microscope is a galvanometer scanned light sheet microscope with a vertically scanned Gaussian laser beam. The images are recorded with a Hamamatsu Orca Flash 4 camera (Hamamatsu Orca Flash4 V2) with a band-pass filter placed in front of Chroma ET525/50m to detect only the fluorescence. The camera exposure time was set to 100 ms per frame, and the excitation laser excitation power was set to @488 nm for GFP. Stacks of 400 planes with a step size of 1 μm were typically recorded.

#### RNA fluorescence in situ hybridization

RNA-FISH based on the HCR on whole-mount inflorescences was performed as previously described (*46*). For cell wall visualization, inflorescence sections were incubated with 0.02% Calcofluor white for 10 min. HCR Gold probe sets were designed and synthesized by Molecular Instruments (https://molecularinstruments.com/). Signal amplification was carried out using HCR Gold amplifiers X2 with label 546 for *GERMIN3* and *GH3.6* transcripts and HCR Gold amplifiers X2 with label 647 to detect *MPL28*. The LSM880 Confocal microscope using a 25x, 0.95-NA water dipping objective. Laser excitations were 561 nm (X2-546) and 633 nm (X3-647).
