# Supplementary material for: Single-nucleus transcriptomics resolves multiple fate dynamics between inflorescence meristem and primary stem
Source: Sci Adv. 2026 Jun 19;12(25):eaee2988. doi: 10.1126/sciadv.aee2988 (PMC13281799; doi:10.1126/sciadv.aee2988)
Supplement: Supplementary file 1 — Figs. S1 to S11 Legends for tables S1 to S16 [file sciadv.aee2988_sm.pdf]

Science Advances
AAAS

Supplementary Materials for

Single-nucleus transcriptomics resolves multiple fate dynamics between inflorescence meristem and primary stem

Sebastián Moreno-Ramírez *et al.*

Corresponding author: Elliot M. Meyerowitz, meyerow@caltech.edu;
James C. W. Locke, james.locke@slcu.cam.ac.uk; Henrik Jönsson, henrik.jonsson@slcu.cam.ac.uk

Sci. Adv. **12**, eaee2988 (2026)
DOI: 10.1126/sciadv.aee2988

The PDF file includes:

Figs. S1 to S11
Legends for tables S1 to S16

Other Supplementary Material for this manuscript includes the following:

Tables S1 to S16

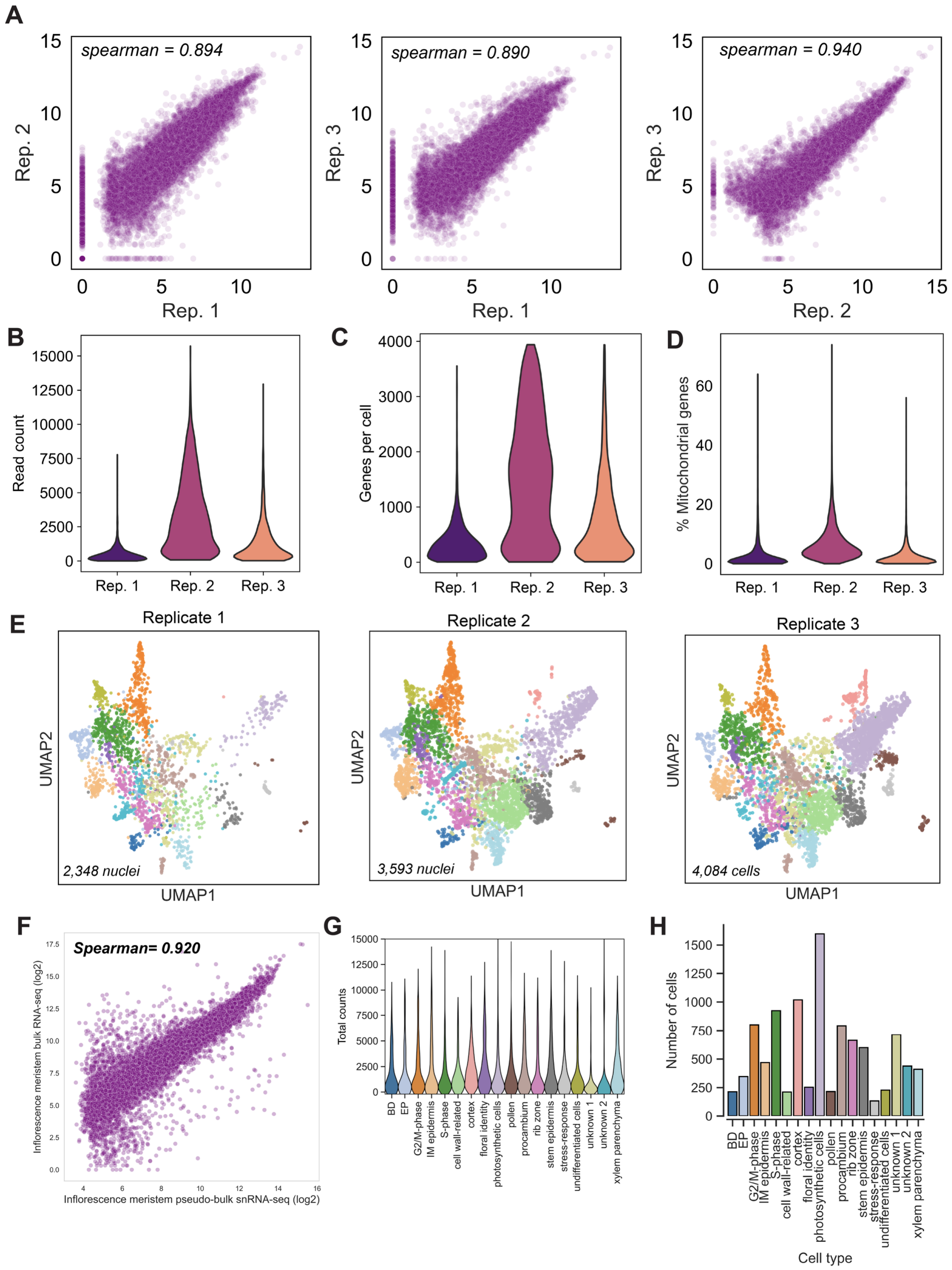

Supplementary Figure 1. Data quality and variability obtained from three independent replicates of wild-type inflorescence meristems. (**A**) Normalized gene expression levels (log_2) between the three different batches used for IM single-nucleus transcriptomic data. (**B**) and (**C**) Violin plot displaying the total number of reads and genes per batch. Mean values are shown at the top of each violin plot. (**D**) Percentage of mitochondrial genes per cell between the different replicates. (**E**) UMAP projection from three independent replicates. Cluster colours are indicated in Sup. Fig. 1G. Number of nuclei filtered after QC per replicate are labelled at the bottom left of each UMAP plot. (**F**) Bulk RNA-seq from inflorescence meristem compared with pooled nuclei from our inflorescence meristem snRNA-seq. Spearman correlation was quantified between the two pooled samples. Three replicates were used for bulk RNA-seq. (**G**) Violin plot illustrating the total number of reads per cluster. (**H**) Number of nuclei per cluster after unbiased clustering analysis.

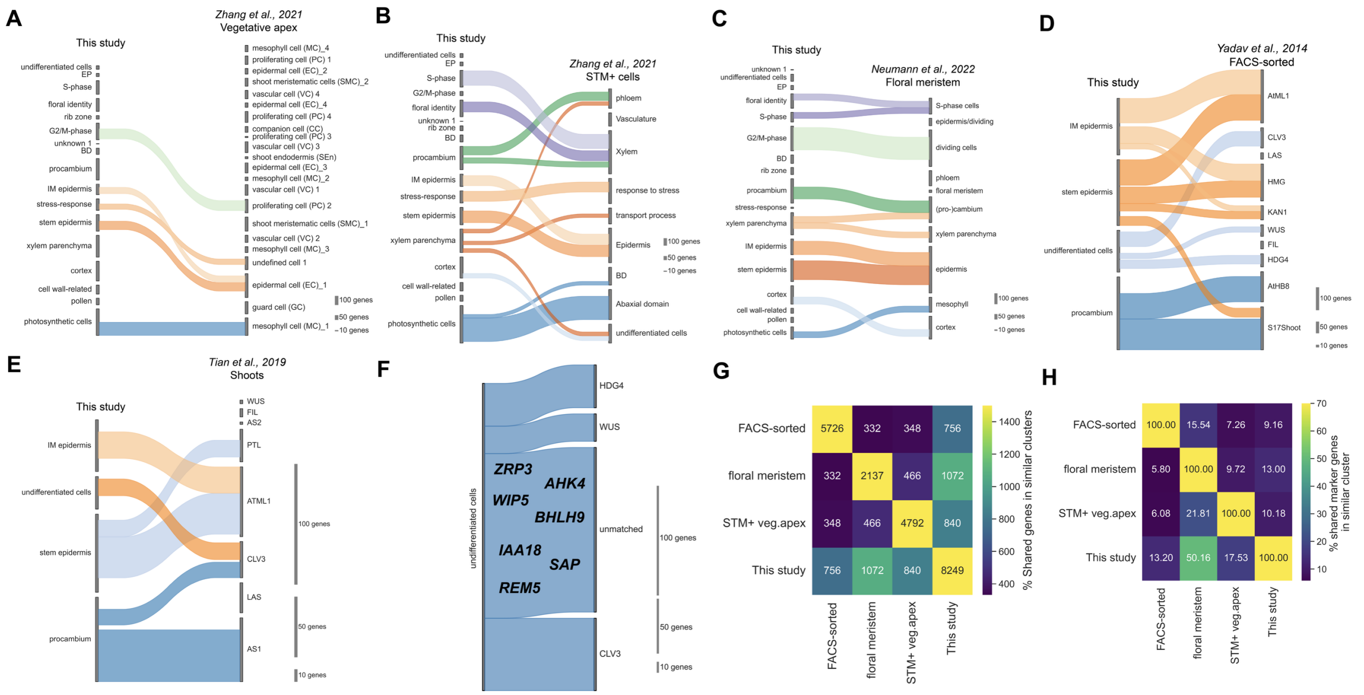

Supplementary Figure 2. Comparison of cell types using marker genes from published apex-related datasets. (**A**) Sankey plot comparing DEGs per cluster from this study and genes obtained in vegetative apex datasets from Zhang et al., 2021(*12*). Links are displayed when ≥ 50 genes are shared. (**B**) Sankey plot comparing DEGs per cluster from this study and genes obtained in STM+ cell population from Zhang et al., 2021(*12*). Links are displayed when ≥ 50 genes are shared. (**C**) Sankey plot comparing DEGs per cluster from this study and genes obtained in floral meristem datasets from Neumann et al., 2022(*22*). Links are displayed when ≥ 50 genes are shared. (**D**) Sankey plot comparing DEGs per cluster from this study and genes obtained in Yadav et al., 2014(*20*). Links are displayed when ≥ 25 genes are shared. (**E**) Sankey plot comparing DEG per cluster from this study and genes obtained in 7 DAG seedlings from Tian et al., 2019(*21*). Links are displayed when ≥ 10 genes are shared. (**F**) Sankey plot comparing DEGs from undifferentiated cell cluster from this study and HDG4+, WUS+ and CLV3+, DEGs in undifferentiated cell cluster that were not detected from FACS-sorted analysis were labelled as unmatched. (**G**) Number of genes shared between different datasets that are expressed in similar meta-clusters. (**H**) Percentage of genes shared between different datasets that are expressed in similar cluster/domains. Percentages are obtained relative to marker genes detected in previously reported dataset.

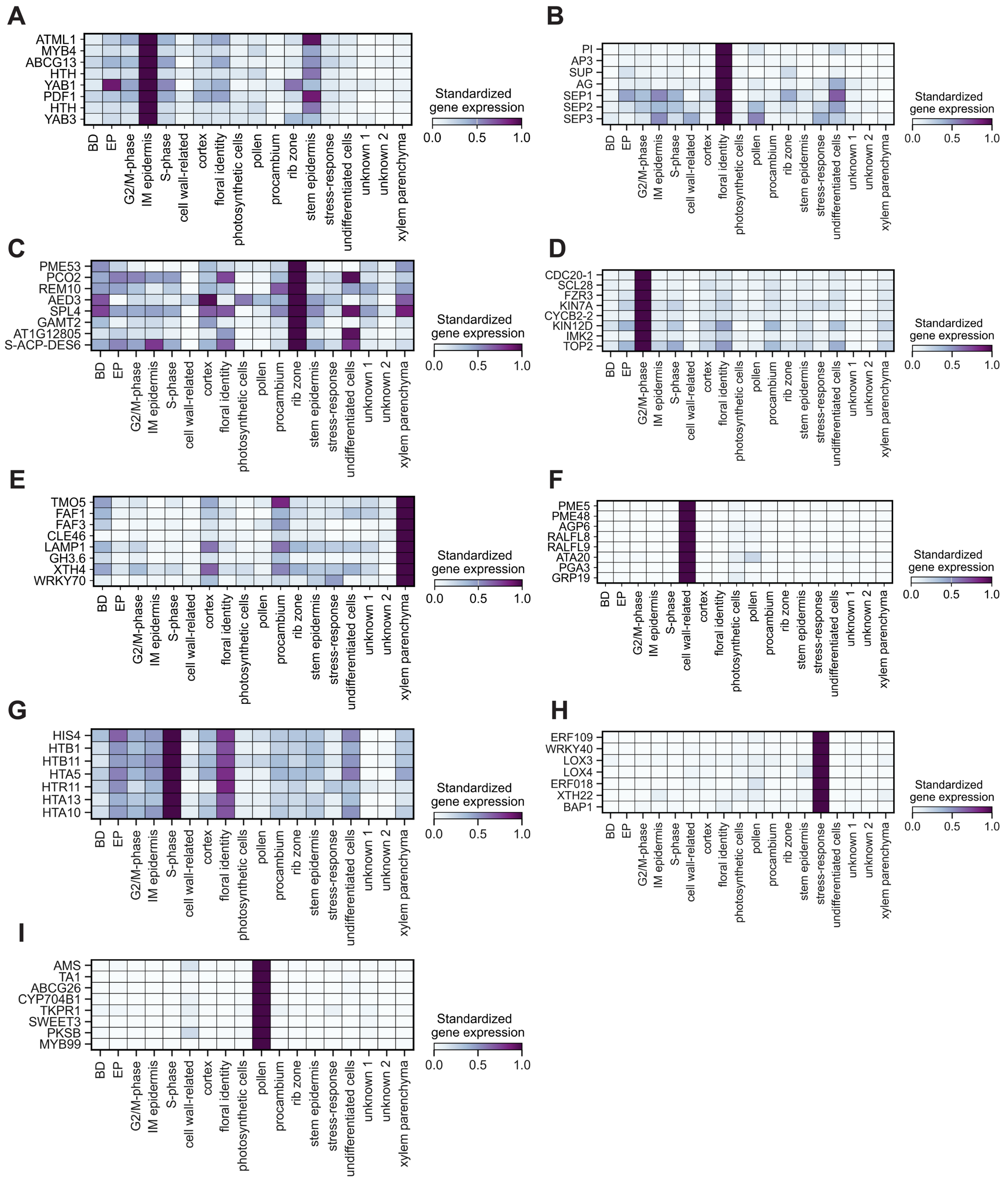

Supplementary Figure 3. Differentially expressed genes per cluster. Heatmap plots illustrating selected marker genes from different clusters. Each gene's expression level across clusters is linearly min-max scaled. (**A**) IM epidermis cluster. (**B**) Floral identity cluster. (**C**) Rib zone cluster. (**D**) G2/M-phase cluster. (**E**) Xylem parenchyma cluster. (**F**) Cell-wall related cluster. (**G**) S-phase cluster. (**H**) Stress-response cluster. (**I**) Pollen cluster.

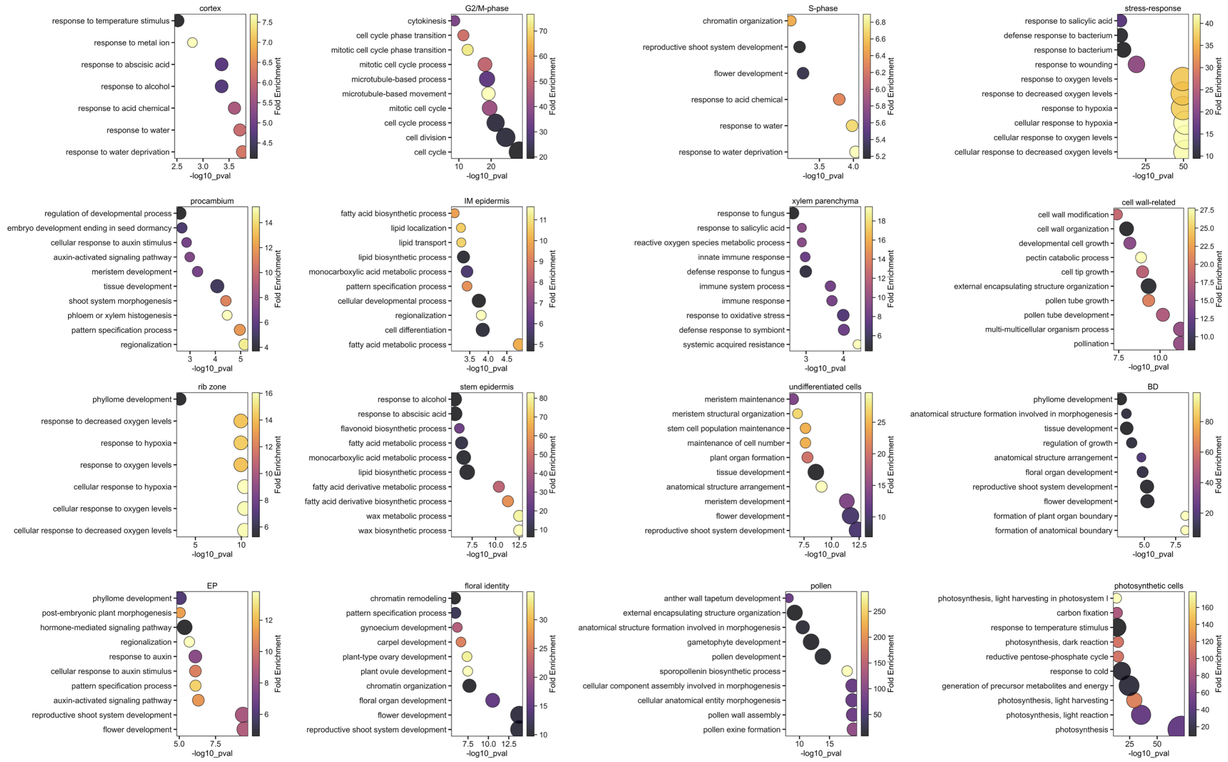

Supplementary Figure 4. Gene Ontology analysis per cluster. Top enriched GO terms per cluster. Fold enrichment is defined as the percentage of genes within the DEG list belonging to a particular pathway, divided by the corresponding percentage in the background. Heatmap indicates Fold enrichment, x-axis indicates -log$_{10}$(p-value) and dot size indicates the number of genes supporting each term.

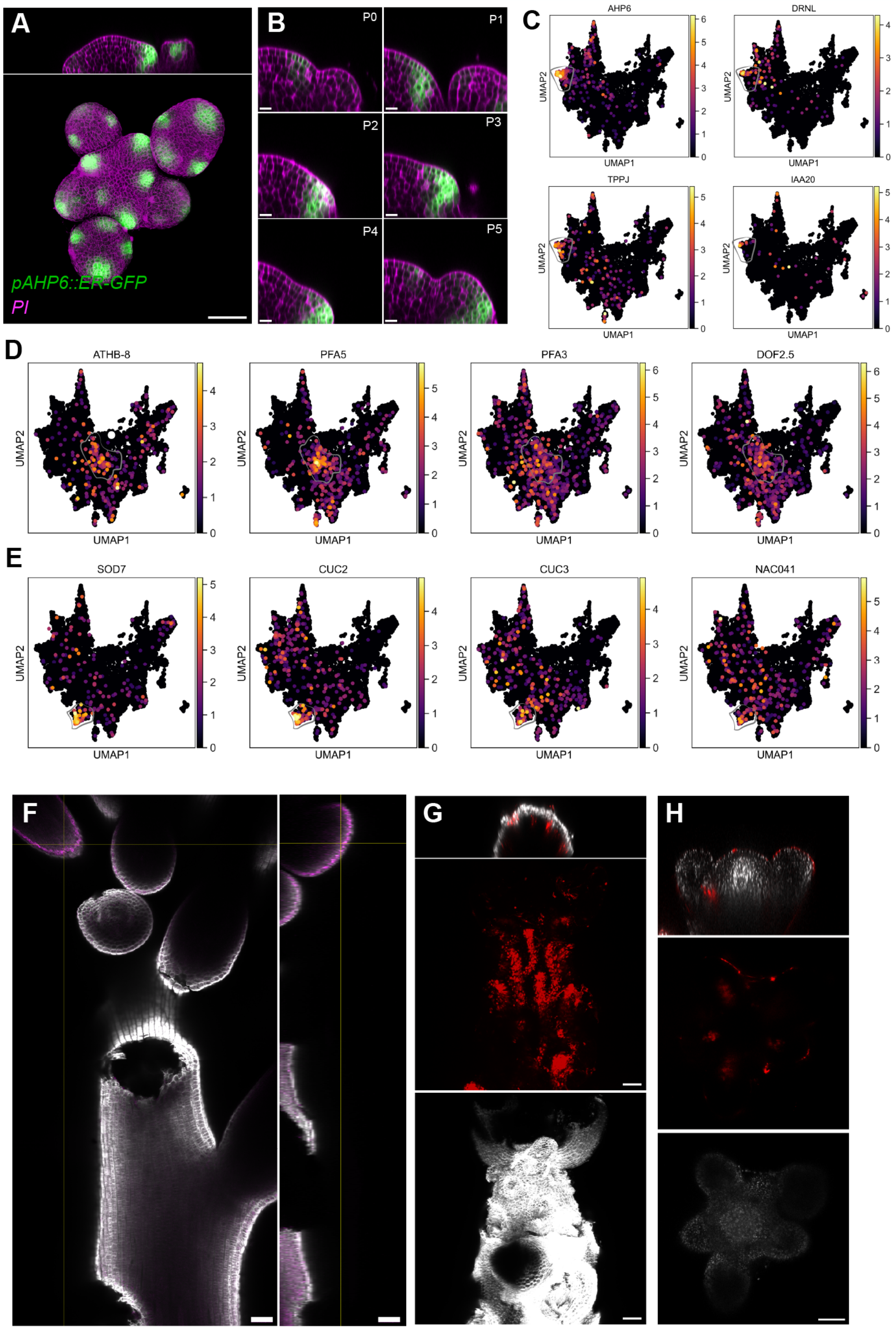

Supplementary Figure 5. **Differentially expressed genes per cluster.** (**A**) Orthogonal projection (top) and maximum projection (bottom) of *pAHP6::ER-GFP* reporter line in the IM. (**B**) Orthogonal projection of *pAHP6::ER-GFP* reporter line following primordium initiation ordered from incipient primordia to primordium number 5. Scale bars, 5 µm. (**C**) UMAP representation of marker genes in EP. The cluster is outlined in grey. (**D**) UMAP representation of marker genes in Procambium cluster. The cluster is outlined in grey. (**E**) UMAP representation of marker genes in the Boundary domain cluster. The cluster is outlined in grey. (**F**) One-slice projection of RNA-FISH showing the *MLP28* transcript in a proximal stem section. (**G**) Orthogonal section of IM and primary stem from *PFA5::H2B-tdTom* reporter line, with the calcofluor white channel shown separately. Merged channels are shown in Fig. 2L. (**H**) Orthogonal section of IM of *PFA5::H2B-tdTom* and chlorophyll channel shown separately.

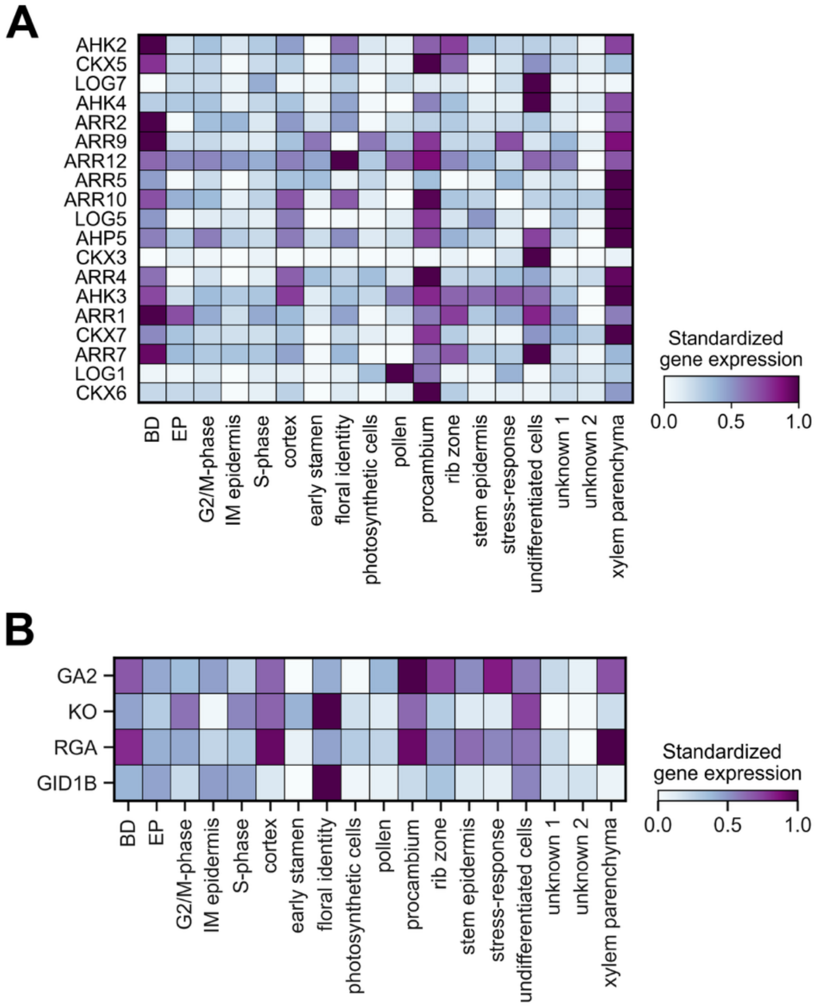

Supplementary Figure 6. Expression patterns of cytokinin and gibberellic acid (GA)-related marker genes per cluster. (**A**) Heatmap plot illustrating standardized expression values of cytokinin-related marker genes. (**B**) Heatmap plot illustrating standardized expression values of GA-related marker genes. Each gene's expression level across clusters is linearly min-max scaled.

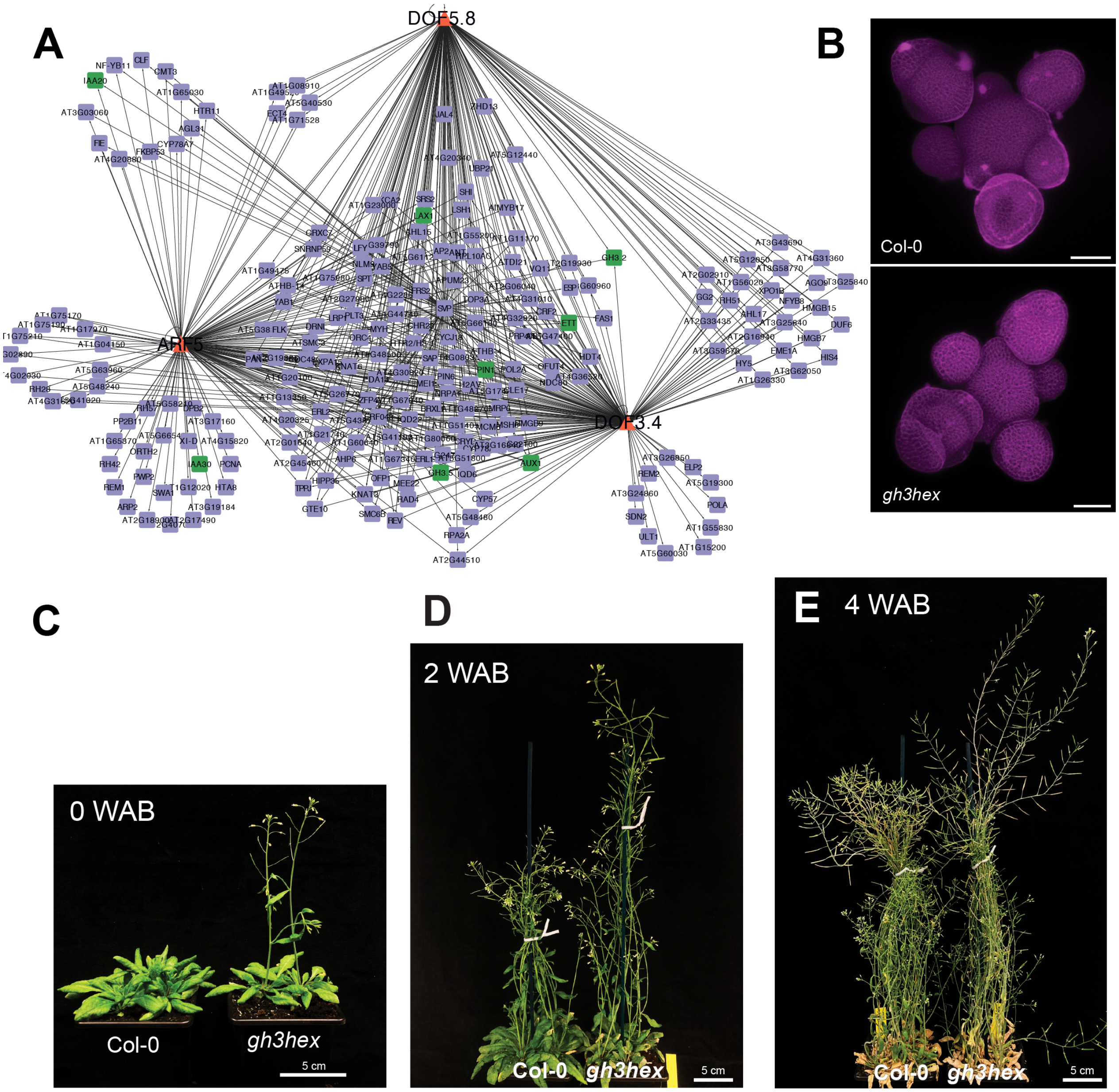

Supplementary Figure 7. The GH3 family is relevant for phyllotactic pattern of siliques. (**A**) GRN of the EP cluster from marker genes identified in the EP cluster. The three most connected TF are shown in red triangles and auxin-related genes are highlighted in green. Target genes are shown in circles. (**B**) Dissected SAM from Col-0 and *gh3hex* mutant lines. Cell walls were stained with propidium iodide. (**C**) to (**E**) *gh3.1/2/3/4/5/6* hextuple mutant (*gh3hex*) and Col-0 plants at different weeks after bolting (WAB).

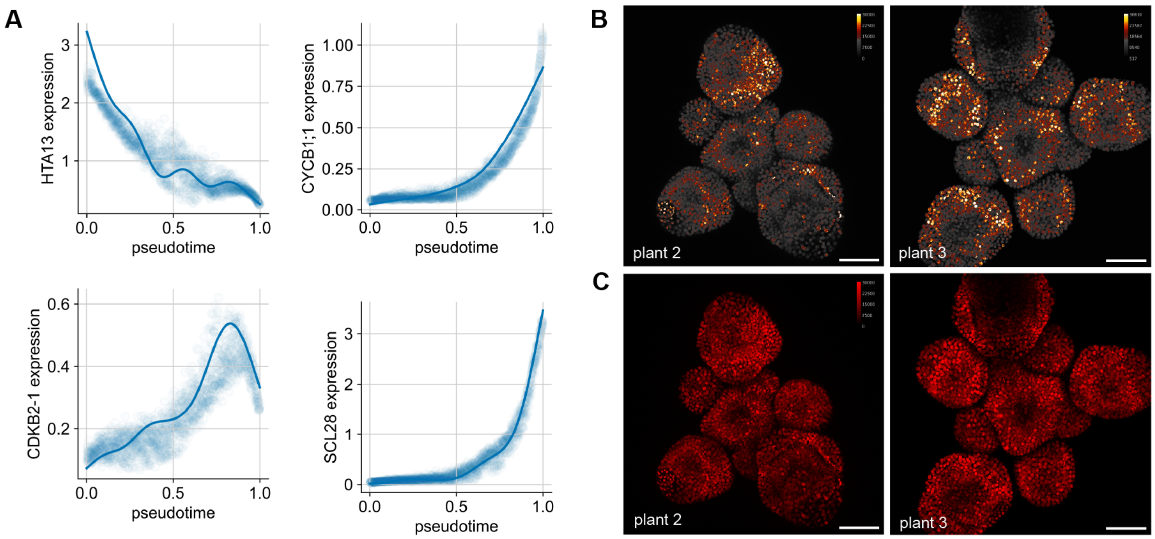

Supplementary Figure 8. Dynamics of differential gene expression along the IM cell cycle. (**A**) Gene expression trends of differentially expressed genes along the cell cycle trajectory. Scatterplots indicate gene expression per cell, and expression trends are depicted with fitted expression levels. (**B**) Maximum projection of SAM from two independent replicates using PlaCCI lines. Fluorescence intensity of the S-phase marker is represented as a heatmap, highlighting regions of higher expression. (**C**) Maximum projection of SAM from two independent replicates using PlaCCI lines, displaying the raw fluorescence intensity of the S-phase reporter line.

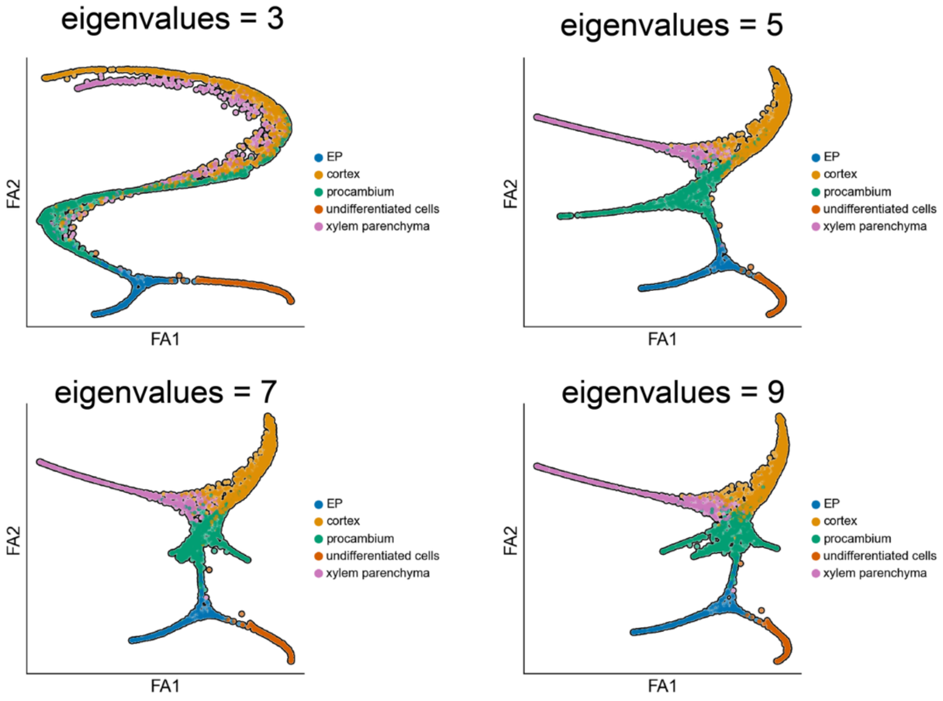

Supplementary Figure 9. Dimensionality reduction analysis for inner cell clusters. Force-directed graph layout of clusters associated with inner cell layers such as EP, undifferentiated cells, procambium, xylem parenchyma and cortex using different eigenvectors. Using nine eigenvalues captured the three different cell identities within vascular bundles from the procambium cluster.

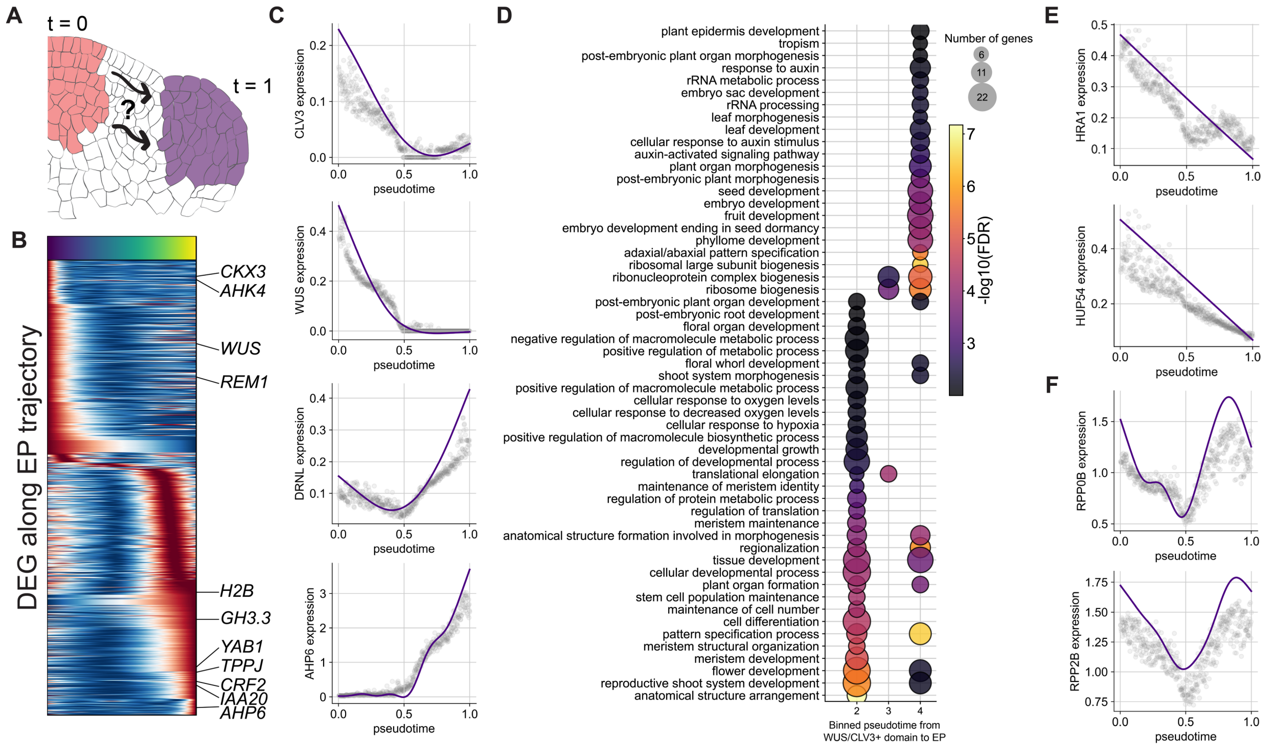

Supplementary Figure 10. Trajectory inference analysis captured dynamic gene expression from shoot stem cells to early primordia. (**A**) Scheme representing the trajectory between undifferentiated cells cluster (pink) and EP cluster (purple). (**B**) Heatmap of DEGs along differentiation from undifferentiated cells to EP. (**C**) Gene expression trends of differentially expressed genes along the EP trajectory. Scatterplots indicate gene expression per cell, and expression trends are depicted with fitted expression levels. (**D**) GO analysis of DEGs along EP trajectory. Genes were ordered by peak expression and sampled into four groups. (**E**) Gene expression trends of DEG related to hypoxia along EP trajectory. (**F**) Gene expression trends of DEG related to translation along EP trajectory.

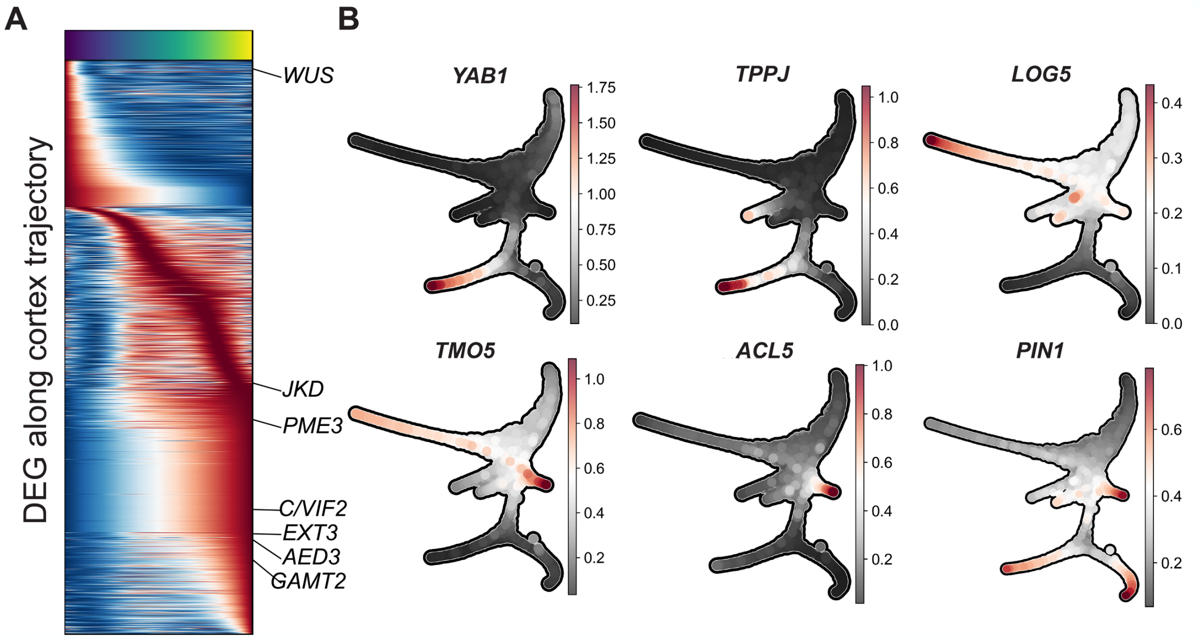

Supplementary Figure 11. Differentiation trajectory of cortex. (**A**) Heatmap of DEGs along early cortex differentiation. (**B**) Heatmap showing expression per cell of *YAB1*, *TPPJ*, *LOG5, TMO5*, *ACL5* and *PIN1* in the force-directed graph layout trajectory.

Supplementary Tables

Supplementary Table 1. QC parameters per replicate.

Supplementary Table 2. Cell-barcode cluster annotation dataset.

Supplementary Table 3. AGI numbers and gene names used in this study

Supplementary Table 4. Differentially expressed marker genes.

Supplementary Table 5. DEGs that are uniquely detected in one cluster.

Supplementary Table 6. GO enrichment analysis per cluster.

Supplementary Table 7. Gene Regulatory Network from EP cluster obtained using DAP-seq.

Supplementary Table 8. Differentially expressed genes along cell cycle trajectory.

Supplementary Table 9. GO ontology analysis of differentially expressed genes along cell cycle trajectory.

Supplementary Table 10. ChIP-seq analysis between floral homeotic genes and cell cycle marker genes.

Supplementary Table 11. Differentially expressed genes along cambium trajectory.

Supplementary Table 12. Differentially expressed genes along phloem trajectory.

Supplementary Table 13. Differentially expressed genes along EP trajectory.

Supplementary Table 14. Differentially expressed genes along xylem parenchyma trajectory.

Supplementary Table 15. Differentially expressed genes along xylem trajectory.

Supplementary Table 16. Differentially expressed genes along cortex trajectory.
